# The Roles of Imaging Biomarkers in the Management of Chronic Neuropathic Pain

**DOI:** 10.3390/ijms232113038

**Published:** 2022-10-27

**Authors:** Cosmin Vasilica Pricope, Bogdan Ionel Tamba, Gabriela Dumitrita Stanciu, Magdalena Cuciureanu, Anca Narcisa Neagu, Ioana Creanga-Murariu, Bogdan-Ionut Dobrovat, Cristina Mariana Uritu, Silviu Iulian Filipiuc, Bianca-Mariana Pricope, Teodora Alexa-Stratulat

**Affiliations:** 1 Advanced Research and Development Center for Experimental Medicine (CEMEX), Grigore T. Popa University of Medicine and Pharmacy, 16 Universitatii Street, 700115 Iasi, Romania; 2 Department of Pharmacology, Clinical Pharmacology and Algesiology, Grigore T. Popa University of Medicine and Pharmacy, 16 Universitatii Street, 700115 Iasi, Romania; 3 Laboratory of Animal Histology, Faculty of Biology, Alexandru Ioan Cuza University of Iasi, Carol I bvd. No. 22, 700505 Iasi, Romania; 4 Department of Radiology, Grigore T. Popa University of Medicine and Pharmacy of Iasi, 16 University Street, 700115 Iasi, Romania; 5 Department of Preventive Medicine and Interdisciplinarity, Grigore T. Popa University of Medicine and Pharmacy, 16 Universitatii Street, 700115 Iasi, Romania; 6 Medical Oncology-Radiotherapy Department, Grigore T. Popa University of Medicine and Pharmacy, 16 University Street, 700115 Iasi, Romania

**Keywords:** neuropathic pain, imaging biomarkers, MRI, PET, MRS, SPECT, MEG, neurostimulation

## Abstract

Chronic neuropathic pain (CNP) affects around 10% of the general population and has a significant social, emotional, and economic impact. Current diagnosis techniques rely mainly on patient-reported outcomes and symptoms, which leads to significant diagnostic heterogeneity and subsequent challenges in management and assessment of outcomes. As such, it is necessary to review the approach to a pathology that occurs so frequently, with such burdensome and complex implications. Recent research has shown that imaging methods can detect subtle neuroplastic changes in the central and peripheral nervous system, which can be correlated with neuropathic symptoms and may serve as potential markers. The aim of this paper is to review available imaging methods used for diagnosing and assessing therapeutic efficacy in CNP for both the preclinical and clinical setting. Of course, further research is required to standardize and improve detection accuracy, but available data indicate that imaging is a valuable tool that can impact the management of CNP.

## 1. Introduction

The first mention of neuropathic pain dates back before the era of modern medicine, as early as the 2nd century AD, when Aretaeus referred, in his neurology manuscripts, to the term neuralgia [1]. Subsequently, the term neuropathy was defined as a nerve disease by R.G. Mayne in 1860, and in 1924, Gordon published an article entitled “Clinical Lecture on Neuropathy”, using, for the first time, the word neuropathy in a medical article indexed in MEDLINE [2]. In the last few decades, however, neuropathy and chronic neuropathic pain (CNP) have become issues of rapidly growing importance, with over 5000 scientific manuscripts published yearly in the field.

The International Association for the Study of Pain (IASP) defines neuropathy as any disturbance of function or pathological change in a nerve. Subsequently, CNP is defined as pain arising as a direct consequence of a lesion or disease affecting the somatosensory system [3]. Current estimates indicate that the prevalence of CNP in the general population is 7.7 ÷11.5% in Canada [4], 8% in the UK [5,6], 6.5% in Germany [7], 6.9% in France [8], 10.6% in Morocco [9], 3.2% in Japan [10], 8.8% in the USA [11], and 10% in Brazil [12]. Perhaps even more important than the increasing prevalence, available data suggest that CNP has a significant impact on a patient’s quality of life, and is strongly associated with conditions such as depression and sleep disturbance. Last, but not least, CNP adds a significant burden to the health system, with increased hospital visits and use of prescription medicine, and has an overall detrimental effect on the economy due to numerous instances of medical leave, decrease in productivity, and subsequent incapacity to work [10,13,14,15,16]. A retrospective cohort study conducted in the UK found that patients with chronic neuropathic pain had an average of 10 additional visits to the family doctor and twice as many hospitalizations, specialist consultations, and sick leave as compared to the control group [17]. Schaefer et al. conducted a study on 624 people diagnosed with CNP, and reported that direct and indirect healthcare costs increased in direct proportion to the severity of symptoms, estimating an average total annual cost of over USD 27,000 per participant [18]. In terms of the cost of losing productivity due to absenteeism in the workplace, Sweden reported, in 2003, approximately SEK 80 billion caused by chronic pain symptoms [19]. In a Canadian analysis, it was reported that the costs of providing medical services to patients with chronic neuropathic pain were double that of the comparator group (CAD 4163 versus 1846 per individual) [20].

Even though it is a pressing healthcare problem that has consequently received increasing research interest [21], CNP remains a condition that is extremely difficult to treat, possibly due to its diverse and non-specific symptoms, or the fact that most underlying mechanisms are still unknown [22,23,24]. It is estimated that only 10–15% of CNP patients will experience a significant benefit (defined as a reduction in pain intensity of at least 50%) using available analgesic drugs [25,26]. Even with the use of non-pharmacological pain treatments, such as implants, the majority of CNP patients will be dissatisfied with their treatment, which will ultimately lead to a significant decrease in the quality of life and numerous hospitalizations. A potential issue and confounder in CNP management is the subjective and heterogeneous manner of diagnosis, which is currently based largely on the questionnaire method. The most commonly used questionnaires are: The Self-Report Leeds Assessment of Neuropathic Symptoms and Signs (S-LANSS), Douleur Neuropathic 4 Questions (DN4), Neuropathic Pain Scale (NPS), and painDETECT. Although they do have reasonable specificity and sensitivity (74% sensitivity and 76% specificity for S-LANSS; 78% sensitivity and 81% specificity for DN4; 85% sensitivity and 80% specificity for painDETECT) [14,27,28,29], using the questionnaire method alone for diagnosing a condition presents serious limitations in vulnerable populations, such as the elderly and demented patients [30]. Additionally, patient-reported outcomes are subjective, which, in turn, may lead to overestimation or underestimation of the presence of neuropathic pain [4].

Therefore, facing a real public health issue and a therapeutic challenge, there is a need to develop new reliable, objective, and quantifiable methods of diagnosing chronic neuropathic pain. This could be achieved by taking advantage of technological advances in medicine, such as the use of combined imaging techniques to detect biomarkers of disease and speed up pharmacological safety and efficacy testing of new compounds in assessing effects and bioavailability through molecular imaging [31,32]. The aim of this paper is to provide both preclinical and clinical researchers and physicians with an overview of the current imaging techniques that offer biomarkers for the diagnosis, prognosis, and efficient management of chronic neuropathic pain.

## 2. Chronic Neuropathic Pain and the Pain Matrix

CNP is a complex syndrome that manifests as a cluster of signs and symptoms. Neuropathic pain is often confused with nociceptive pain, both in the clinical and in the preclinical setting. While nociception occurs when non-neuronal tissues are injured, neuropathic pain necessarily involves damage to the components of the somatosensory nervous system [33]. The irritation of the sensory nerves triggers a cascade of events, transmitting painful signals at different levels that ultimately leads to the production of a painful sensation. The normal circuits of pain sensation begin at the skin, muscle, or visceral level, where specific receptors respond to the painful stimulus by creating a depolarization wave from the activation of Na/K pumps. The electrical impulse is transmitted to the spinal cord (dorsal horn), where the synapse with the first-order neuron takes place. At this level, more specifically, in the presynaptic cleft, the dependent voltage-gated Ca channels are activated, and the Ca influx allows the release, into the synaptic space, of excitatory neurotransmitters (glutamate). These neurotransmitters bind with specific receptors, located at the level of the second neuron, thus triggering its depolarization. The nerve fibers rise upwards, intersect, and reach the third synapse in the thalamus, so that later the dendritic extensions reach the limbic system and the cerebral cortex, where the information contributes to the sensation of pain [34].

Physiologically, each painful stimulus is accompanied by subsequent inhibition, which is most often the responsibility of antinociceptive neurons located in the brainstem. The extensions of these neurons descend to the spinal cord and connect with short interneurons in the dorsal horn, leading to the secretion of serotonin and norepinephrine. Interneurons modulate the synapse between first-order neurons and second-order neurons by releasing an inhibitory neurotransmitter, GABA. Hence, pain cessation is the result of the inhibition of synapses between first- and second-order neurons, while pain enhancement may be the result of suppression of inhibitory synaptic connections [34].

In the case of neuropathic pain, damage to the somatosensory nervous system, either through injury or disease, can lead, through various mechanisms, to a state of hyperexcitability. Injuries to the peripheral nerves, dorsal root ganglion (DRG) or dorsal root, or the central nervous system lead to clinical syndromes, some with “negative” symptoms or sensory deficits such as partial or complete lack of sensation, and others with positive symptoms such as dysesthesia, paresthesia, and pain [35].

### 2.1. Classification and Etiology

Currently, there are several classifications used for chronic neuropathic pain. In the clinical setting, physicians most often discriminate between different neuropathies based on etiology, pain characteristics, and the anatomic location of the lesion. Although useful for the differential diagnosis, these classifications do not necessarily reflect a different management course or distinct response to treatment.

Woolf and Mannion suggested classifying neuropathic pain according to the presence/absence of the painful stimulus, i.e., stimulus-independent pain and stimulus-evoked pain. Stimulus-independent pain can further be divided into continuous and paroxysmal pain [35]. In stimulus-evoked pain, two key features must be taken into account: hyperalgesia and allodynia. Hyperalgesia is an exaggerated response to a stimulus that would normally cause pain, being an abnormal processing of a nociceptor stimulus. It can be caused by mechanical, thermal, or chemical inputs. Mechanical hyperalgesias are further classified as brush-evoked (dynamic), pressure-evoked (static), and punctate hyperalgesias. On the other hand, allodynia appears when a normally innocuous stimulus triggers pain, having two known mechanisms: by the action of low-threshold myelinated A fibers on an altered central nervous system, or by a reduction in the threshold of nociceptor terminals in the periphery [35].

Chronic neuropathic pain can be a symptom of several conditions, which is why it is commonly divided into categories: peripheral, central, or mixed. Whereas central neuropathic pain is a consequence of injuring sensory fibers belonging to the central nervous system (brain and spinal cord), peripheral neuropathic pain results from damage to the peripheral nerve fibers and originates in small non-myelinated C and F-type myelinated fibers (Aβ and Aδ). The injury of these nerve fibers leads to changes in the expression of neurotransmitters, neuromodulators, growth factors, receptors, neuroactive molecules, and inflammatory mediators, thus leading to hypersensitivity in reaction to stimuli [36]. Chronic neuropathic pain with peripheral etiology can be further divided into generalized (polyneuropathies) and focal or multifocal neuropathies.

### 2.2. Structural and Functional Changes in CNP

In the case of neuropathic pain, the mechanisms are not fully known and the relationship between etiology, mechanisms, and symptoms is extremely complex [37]. A single mechanism may be responsible for producing several symptoms, and the same symptom may be caused by different mechanisms from one patient to another. No pain mechanism is an inevitable consequence of a particular disease process, and there are no consistent predictors to indicate which patient will develop neuropathic pain [35].

Available literature data indicate there are at least six different mechanisms involved in the chronicity of neuropathic pain: increased activity in the brain areas that make up the “pain matrix”, recruitment of additional cortical areas beyond the pain matrix, cortical reorganization and maladaptive neuroplasticity, structural brain changes, disruption of the brain default mode network, and alterations in neurochemistry [38].

#### 2.2.1. Increased Activity in the Pain Matrix

**Figure 1:**
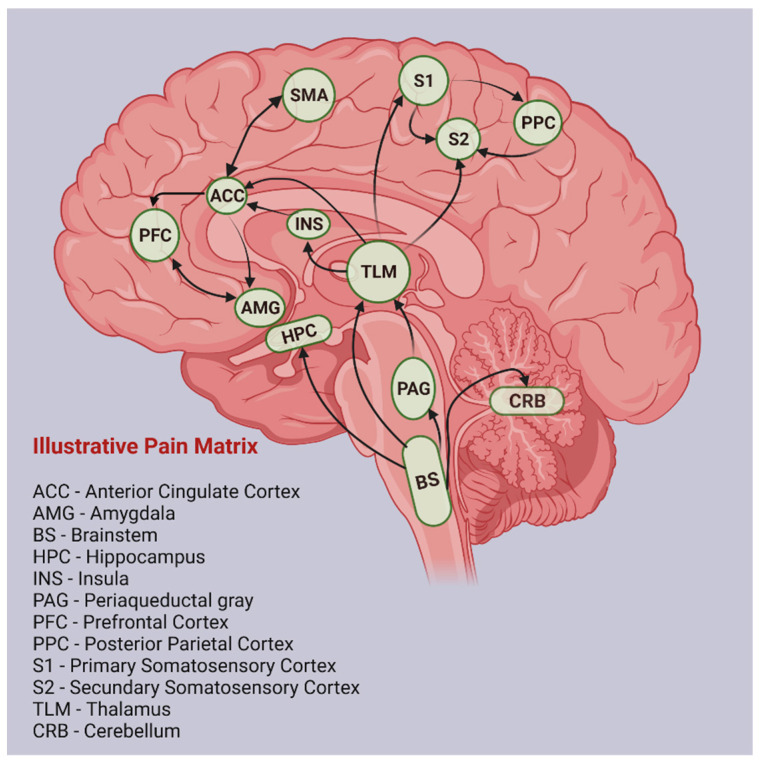
Illustrative pain matrix (created with BioRender.com, accessed on 15 March 2022). ACC = Anterior Cingulate Cortex; AMG = Amygdala; BS = Brainstem; HPC = Hippocampus; INS = Insula; PAG = Periaqueductal gray; PFC = Prefrontal Cortex; PPC = Posterior Parietal Cortex; S1 = Primary Somatosensory Cortex; S2 = Secundary Somatosensory Cortex; SMA = Supplementary Motor Area; TLM = Thalamus; CRB = Cerebellum.

Several studies have shown that at the cortical and subcortical levels, there is a complex network involved in various aspects of the perception of painful sensations produced by thermal, mechanical, or chemical stimuli: the so-called “pain matrix” [39,40] (Figure 1). By means of functional imaging techniques, the following structures have been identified as playing a key role in the pain matrix: the thalamus, the primary and secondary somatosensory cortex (S1, S2), the insular cortex, the anterior cingulated cortex (ACC), and the prefrontal cortex (PFC). The sensory–discriminative aspect of pain is associated with the S1, S2, thalamic nuclei, and the posterior insula, known as the lateral pain system. The affective–motivational processing of pain, which makes up the median pain system, has been associated with ACC, anterior insular cortex, PFC, and thalamic nuclei. Finally, the cognitive aspects of pain are considered to be closely related to PFC [41].

Increased activity in the pain matrix secondary to CNP has been strongly endorsed by modern imaging techniques (see section three of the article), although changes in the CNS vary greatly depending on the type of condition and its symptoms. For example, studies have indicated changes in contralateral thalamic activity with concurrent decrease in thalamic blood flow in patients with unilateral CNP [42]. However, some CNP conditions, such as mechanothermal allodynia, can be associated with increased thalamic activity [43].

In addition to these changes, the literature states that the existence of synaptic changes in the brain structures is responsible for modulating the perception of pain. Thus, long-term cognitive and mood changes associated with neuropathic pain may be caused by synaptic changes that occur in the amygdala, anterior cingulate gyrus, and prefrontal cortex [44,45].

#### 2.2.2. The Implication of Additional Cortical Areas

Changes in brain regions outside of the pain matrix as a response to chronic neuropathic pain, commonly referred to as a “pain signature”, can involve cortical areas normally responsible for attention, affection, and mood. These changes are unique to each patient, and most commonly affect the dorsolateral frontal cortex, areas of parietal association, and some of the brainstem nuclei embedded in pain modulatory systems [46].

#### 2.2.3. Cortical Reorganization and Maladaptive Neuroplasticity

Although brain reorganization and maladaptive neuroplasticity are noted in several conditions that lead to CNP, phantom pain syndrome is probably the best researched. This type of neuropathic pain occurs after amputation of a limb, and is characterized by a variety of sensations, including the feeling of the presence of the amputated extremity and paresthesia inside the phantom member. Magnetoencephalography (MEG) imaging was performed in patients with this condition, and it was observed that the distribution area of the mouth in the somesthetic area was found to be shifted into that of the former limb. The extent of the shift was directly proportional to the intensity of the perceived neuropathic pain [47].

The pathophysiological basis of neuroplasticity in neuropathic pain could be based on the fact that the stimulation of nociceptors on the limb, before it is amputated, produces some changes in the brain, thus creating a memory area of this type of pain. Thus, maladaptive plasticity is a very likely explanation for the perception of pain in the phantom limb [48].

#### 2.2.4. Structural Brain Changes

Several types of changes in both gray and white matter have been reported in patients with CNP. A study by Jutzeler and coworkers assessed patients with traumatic spinal cord injury, comparing by means of clinical and imaging assays those that developed CNP versus those that were pain-free. The authors concluded that CNP was associated with smaller cord area, increased gray matter in the left anterior cingulate cortex and right primary motor cortex, and decreased gray matter in the right primary somatosensory cortex and thalamus. Similarly, another study assessed changes in the brain structure of patients that had undergone unilateral limb amputation, and found that phantom limb pain was correlated with a decrease in gray matter density in the PFC, SMA, dorsal midbrain, and cingulate cortex, which was directly proportional to the intensity of the pain [49].

#### 2.2.5. Disruption of the Default Mode Network

The default mode network is a system of connected brain areas that show increased activity when the person is not focused on what is happening around them [50]. In the case of chronic neuropathic pain, some connections in this network appear to be altered, a feature objectified by fMRI in a group of patients suffering from chronic back pain. While performing a visual task, these patients experienced reduced deactivation in several key default mode network regions, compared to the control group, suggesting that network damage could be the cause of the behavioral and cognitive changes that accompany chronic pain [51].

#### 2.2.6. Alterations in Neurochemistry

In the pathophysiology of chronic neuropathic pain, peripheral sensitization plays a key role, and is generated by the appearance of perilesional inflammatory changes. The secretion of mediators (e.g., neurotrophic growth factor, PGE2, bradykinin, cytokines, and chemokines) leads to the stimulation of the axons of peripheral neurons, and thus the activation and migration of macrophages in the nerves and dorsal root ganglion. Inflammatory mediators interact with cellular receptors and ion channels, leading to a series of intracellular changes, ultimately decreasing activation thresholds and increasing membrane excitability, which consequently results in a painful sensory effect [52]. Another consequence of neurochemical changes secondary to nerve injury is the activation of other neural cells located in the vicinity. These cells can release immune modulators that paradoxically promote nociception by altering neuronal function. For example, in the presence of adenosine triphosphate (ATP), microglia can increase the production and release of signaling molecules, such as proinflammatory cytokines and brain-derived neurotrophic factor (BDNF), which can modulate the activity of neurons, including pain-transmitting dorsal horn neurons, thus playing a significant role in the maladaptive plasticity of the nervous system [53].

### 2.3. Biochemical Changes in CNP

Understanding the long-term modifications of the peripheral and central nervous system after chronic pain has greatly increased in the past few years due to emerging preclinical research techniques, mostly performed in vitro. Such approaches are useful in understanding sensory neuron functions, based on their sensory and nociceptor-specific molecular profiles [54], i.e., ion channels and neuropeptides. The best-known ion channels involved in CNP are the voltage-activated sodium channels (Na_v_) [55], the voltage-gated calcium channels (Ca_v/_VGCC), the calcium-activated potassium channels (K_Ca_), the purinergic receptor (P_2_X), the transient receptor potential vanilloid family ion channel 1 (TRPV1), and the transient receptor potential cation channel ankyrin 1 (TRPA1) [56]. Of the neuropeptides, calcitonin-gene-related peptide (CGRP), substance P (SP), galanin, somatostatin and its receptors, endothelin-1 (ET1), angiotensin II and its receptors, isolectin B4 (IB4), neurotrophins, nitric oxide synthase (NOS), gamma amino butyric acid (GABA), and phospholipase β3 are known as molecules characteristic of nociceptors [57].

Spinal glial cells, such as microglia or resident macrophages of the CNS, astrocytes, and oligodendrocytes [58] have a large impact on the neuropathic pain condition [59], enhancing excitability of spinal dorsal horn neurons, which results in pain amplification and distortions [60]. Thus, rat microglia cell lines are also cultured, transfected, or stimulated [61], as well as neuron-free satellite glial cell (SGC) cultures that allow the fluorescent immunolabelling and analysis of biomarkers expression. For example, TRPA1 has been identified by immunocytochemistry (ICC) both in SGC dissociated culture coexisting with glial fibrillary acidic protein (GFAP), as well as in neurons in DRG, by immunohistochemistry (IHC), and within acute DRG dissociated culture, where TRPA1 coexists with the pan-neuronal marker β3-tubulin (Tubb3) [56]. GFAP, a glial cell biomarker important for tissue maintenance, remodeling, and plasticity, was detected by IHC as being overexpressed in the satellite cells, the major glial cells surrounding DRG neurons, where they play important roles in the development and modulation of chronic pain [62] in experimentally injured rat DRG [63].

The data indicate that peripheral nerve injury induces an overexpression of sensory-neuron-derived colony-stimulating factor 1 (CSF1) (also known as macrophage colony-stimulating factor (M-CSF) [64]), matrix metalloproteinase-9 (MMP-9), neuregulin-1 (NRG1), and caspase-6 (CASP6) in the injured DRG neurons [65]. CSF1 is a growth factor involved in the differentiation and proliferation of macrophages in DRG and microglia in the CNS that interacts with its protein tyrosine kinase CSF1R receptor expressed in microglia in the brain and spinal cord [64]. IHC has demonstrated that damaged DRG neurons show an increase in CSF1 levels, together with an increase in the concentration of the activating transcription factor (ATF3) protein [66], linked to the regenerative response in both motor and sensory neurons after nerve root injury [67]. Nerve injury induces CSF1 overexpression in both injured DRG sensory neurons and in ventral horn motoneurons [68]. MMP-9 is detected in Schwann cells hours after peripheral nerve injury, controlling axonal degeneration and macrophage recruitment to the lesion [69]. The upregulation of MMP-9 and MMP-2 in DRG and the spinal cord is believed to contribute to the pathogenesis of chronic pain [70]. The inflammatory mediator MMP-9 is involved in the degradation of the extracellular matrix (ECM), helping the immune cells to migrate to the inflammation sites [71]. MMP-9 induces neuropathic pain via interleukin-1β (IL-1β) cleavage and microglial cell activation at early stages, whereas MMP-2 maintains neuropathic pain through IL-1β cleavage and astrocyte activation at later stages [72]. MMP-9 gene deletion reduced unstimulated neuropathic nociceptive behavior and preserved myelin thickness by protecting myelin basic protein (MBP) from degradation [69]. NRG1 is a growth and differentiation factor released after nerve injury that drives microglial proliferation, survival, and motility. NRG1 is associated with a proinflammatory phenotype, and can lead to neuropathic pain through the mitogen-activated ERK-regulating kinase (MEK)/ERK pathway in microglia [73]. Major histocompatibility complex (MHC) II β immunostaining revealed an increased number of NRG1-positive cells present in injured DRG [63].

## 3. Current Imaging Techniques Used in CNP

### 3.1. Structural and Functional Imaging for CNP Diagnosis

Several imaging technologies are currently used for providing objective structural and functional measurements of different brain regions involved in the perception of pain. The best-known imaging tools are magnetic resonance imaging (MRI), positron emission tomography (PET), single-photon emission computed tomography (SPECT), electroencephalography (EEG), and magnetoencephalography (MEG) [74].

MRI is a versatile technique that produces cross-sectional high-resolution images using a strong magnet and radio waves. From an anatomical point of view, gray matter can be assessed by MRI scanning with voxel-based morphology (VBM) or cortical thickness analysis (CTA). On the other hand, the changes caused by neuropathic pain in white matter are visible by means of MR-based diffusion tensor imaging (DTI) [75]. MRI has been used for several years in patients with chronic neuropathic pain, especially in cases that could benefit from surgery. A recent analysis estimated that MRI-based biomarkers have the ability to predict the success of pain-alleviating surgery with 90% sensitivity and 66% specificity [76]. Using VBM and extracting brain gray matter density from magnetic resonance imaging, Ung and coworkers compared data for chronic lower back pain patients and healthy controls. The authors reported an average accuracy of 76%, with positive and negative predictive values of 75% and 76%, respectively [77]. Similar data were obtained by Labus and coworkers for patients with irritable bowel syndrome, with a predictive accuracy of 70%. However, despite good specificity and sensibility, the main caveat of classical MRI imaging is that it can only offer a two-dimensional view of the brain, and a significant portion of the changes induced by CNP are functional. As such, the validation of functional MRI (fMRI) in the early 1990s has greatly contributed to the field of pain research. This tool is used for studying both sensory processing and the control of action [78]. Additionally, it can identify functional changes that depend on blood oxygenation in the brain at rest or the activation of various cortical areas by means of assessing the blood-oxygenation-level-dependent (BOLD) signal [79]. Available literature data indicate that in the case of neuropathic pain, there is a specific pattern that is visible via fMRI. A study performed by Baliki and coworkers assessed patients with chronic back pain, and analyzed the different stages of spontaneous neuropathic pain using fMRI. In phases of increasing pain, the activation of the classic areas of the pain matrix was objectified. In contrast, during phases of high spontaneous pain, the increased activity was located in the PFC and ACC, suggesting that subjective pain involves distinct spatiotemporal neuronal mechanism, different from those observed during acute experimental pain [80]. Available data regarding the discriminatory abilities of fMRI are encouraging. A study involving patients with chronic lower back pain used resting state fMRI to discriminate them from healthy controls, and reported an overall accuracy of 68% [81]. More recently, magnetic resonance spectroscopy (MRS) has also emerged as an MRI-derived imaging tool with applications in CNP. MRS can determine the concentration of different metabolites in brain tissue, and is particularly useful for assessing neuroinflammation and neurodegeneration [82]. As such, MRS provides a different perspective of the analyzed tissue, showing local metabolic changes through the accurate spectral graph variations of chemical composition [83]. Last, but not least, MRI-based techniques can be used for localizing inflammation during nerve repair processes. After the administration of iron oxide nanoparticles, a compound that is internalized by macrophages, MRI can identify the presence of the compound inside macrophages, and thus highlight the site of inflammation due to the superparamagnetic properties of iron [84].

PET and SPECT are molecular imaging techniques with high sensitivity and good spatial resolution and penetration depth that have made a significant contribution in the evaluation of the physiological function and biochemical changes of molecular targets. Both techniques are based on the quantification of radionuclide decay, during which a positron or a γ-ray is detected. One of the main advantages that makes these imaging technologies vital, both for preclinical and clinical studies, is their capability of using highly specialized radiopharmaceutical probes tailored for specific indications, without changes in the chemical structure of the ligand [85]. Imaging studies have evaluated the changes in the brain induced by spontaneous neuropathic pain, reporting sensitivity scores of 2.37–7.02 for SPECT imaging [86]. PET allows the measurement of cerebral blood flow, making it possible to compare activated/deactivated brain regions with the onset of neuropathic pain. For example, studies have shown a decrease in cerebral flow at the thalamic level, contralateral to the region where the nociceptive input was located. In contrast, the ACC and PFC showed an increase in cerebral flow, objectified by PET [87]. Similarly, both PET and fMRI have shown that different subtypes of allodynia (cold allodynia, dynamic tactile allodynia, etc.) activate different cortical areas. For example, dynamic mechanical allodynia led to the activation of the lateral pain system (S1, S2), via the insula, parietal, and frontal cortices, without producing changes in the ACC. In contrast, hyperalgesia has been reported to lead to substantial changes in the pain matrix [38].

EEG and MEG are electrophysiological imaging techniques that create electrical brain wave maps of various areas with high temporal resolution, but low spatial resolution and specificity. EEG collects the impulse of neural electric activity of a specific region with the aid of scalp electrodes, and MEG maps the brain activity by recording magnetic fields produced by electrical currents spontaneously occurring in the brain, using highly sensitive magnetometers [88].

There is a significant body of literature data referring to the application of these functional and structural techniques for diagnosing CNP. Over time, research on neuropathic pain has focused on spontaneous pain (paroxysmal and/or ongoing) or evoked pain resulting from painful stimulation, pin-prick, thermal hyperalgesia, hyperalgesia mechanical allodynia, or thermal allodynia [41,89,90,91]. A summary of relevant articles in the field, and their findings, can be found in Table 1.

### 3.2. Imaging Techniques for Assessing Pain-Relieving Implants

In the context of a high number of patients that respond poorly to conventional analgesic treatment, a wide range of non-pharmacological therapies are used for managing CNP [120]. Of these, procedures that rely on neurostimulation are of particular interest due to good results and potential future applications in the field of chronic pain. The main invasive neurostimulation techniques used in the treatment of chronic neuropathic pain are: deep brain stimulation (DBS), motor cortex stimulation (MCS), spinal cord stimulation (SCS), peripheral nerve stimulation (PNS), and nerve root stimulation (NRS) [121,122]. The main non-invasive neurostimulation techniques are transcutaneous electrical nerve stimulation (TENS) and repetitive transcranial magnetic stimulation (rTMS).

Deep brain stimulation can be used to manage therapy-refractory chronic pain using an electrode inserted into subcortical brain structures under local anesthesia, connected to a subcutaneous implantable pulse generator (IPG), often placed in the chest region [123]. DBS is currently used for patients suffering from peripheral neuropathic pain, trigeminal neuropathic pain, phantom limb pain, and central pain syndromes [124,125].

According to the findings of a meta-analysis [126], DBS is significantly more beneficial for nociceptive pain than for neuropathic pain (63% versus 47% in long-term pain management). In terms of CNP, the reported results are heterogeneous. While one study found that somatosensory thalamus or PAG/PVG stimulation resulted in more than 30% pain relief for 67% of patients with central post-stroke pain [124], another found that DBS was ineffective in patients with various neuropathic pain conditions, with only 24% of patients maintaining long-term pain control [127].

Spinal cord stimulation entails implanting electrodes into the epidural space of the cervical or dorsal spine, which can be introduced percutaneously while under local/general anaesthesia, or in open procedure for plate electrode systems. SCS may be beneficial in treating different ischemic and neuropathic pain disorders. One clinical trial on failed back surgery syndrome found that SCS is more beneficial than surgical reintervention [128], while others [129,130,131] found that it is more successful than conventional medical therapy alone, with pain reduction >50% in 48% in the SCS-treated group against 12% in the controls.

In the dorsal root ganglion stimulation procedure, the wire is placed precisely next to the spinal ganglion (one dermatome); therefore, in order to cover a larger region of discomfort, more than one electrode has to be implanted. Under DRG stimulation, a study group with complex regional pain syndrome (CRPS) exhibited a 62–82% reduction in pain intensity levels, suggesting that in monoradicular pain disorders, or pain syndromes involving a small number of dermatomes, DRG stimulation may be an effective option [132,133,134].

Motor cortex stimulation entails implanting one or two epidural electrodes, either parallel or orthogonal to the central sulcus, in the contralateral motor cortex of the painful area, through frontoparietal craniotomy. [135,136] In contrast, in the peripheral nerve stimulation procedure, electrodes are implanted percutaneously to make direct contact with the nerve suspected to be the source of the neuropathic pain (e.g., the main nerves of the limbs, trigeminal, occipital, or facial nerve branches) [129,137,138].

Non-invasive neurostimulation techniques, such as transcutaneous electrical nerve stimulation, entail placing surface electrodes on the skin that covers the painful region, or on the pathway of the nerve that innervates it. TENS is recommended for individuals who have an intact Aβ fiber pathway and whose pain is localized to a relatively small region or territory innervated by an easily accessible nerve. Because it is non-invasive, this type of stimulation is easily accepted by the patient, and may thus be utilized as an adjunct therapy to pharmaceutical treatment or other physical therapies [139].

Repetitive transcranial magnetic stimulation is accomplished by placing a coil of a magnetic stimulator on the scalp over a particular cortical area, with the goal of obtaining analgesic benefits by non-invasive cortical stimulation in patients with chronic pain [140]. Because the efficacy is small and temporary, rTMS should not be used as the only therapy for persistent neuropathic pain. This approach may be offered for short-term analgesia or to identify eligible patients for an epidural (MCS) implant [121,141].

In the field of pain-relieving implants, imaging plays an essential role. Various techniques can provide information not only about the implant position and postoperative local adverse effects (hemorrhage, infection, fibrosis, etc.) caused by implants, but can detect measurable structural and molecular changes in response to a neuroimplant stimulus. These changes may be seen as imaging biomarkers that can provide clues towards a better understanding of the pathogenesis of neuropathic disease, and may generate hypotheses to explain why it appears that neurostimulation is not always effective in terms of pain alleviation. Table 2 provides a brief summary of the available data in the field, emphasizing the imaging method used and the CNS changes secondary to implant use.

## 4. Emerging Techniques for Diagnosing CNP

All of the imaging investigations discussed so far offer low specificity and sensitivity for the etiologic diagnosis of neuropathic pain. It is also important to note that the diagnosis of chronic neuropathic pain is primarily based on patient symptoms, and will probably remain so in the near future. Structural and functional changes in the CNS or the peripheral nervous system do not always correlate with the presence or absence of neuropathic pain, and there is no link between the severity of the alterations and the intensity of perception. Moreover, there is still not enough data to clearly state positive and negative predictive values for any of the imaging tests discussed in this article, most likely due to several factors: heterogeneity of clinical trial inclusion criteria, interobserver variability, technical performance of the device, different chronic pain models, and various CNP etiologies in clinical practice [41]. These important hindrances could have a significant impact on patients’ lives and lead to unnecessary exposure to expensive and potentially irradiating imaging tests [155]. As such, improving specificity and sensibility for any CNP imaging assessment considered for clinical use should be a priority, especially since some of the available imaging techniques can often link CNP to changes in the nervous system, thus aiding the diagnostic process. Of note, improvements in nerve structure or function can sometimes be an indicator that the treatment is, or will be, effective [155]. Moreover, imaging biomarkers will probably be used more and more for predicting which patients will develop CNP after a nerve lesion, and for identifying those at risk of CNP, which is particularly useful when choosing a neurotoxic treatment (as is the case with cancer patients and chemotherapy). In this sense, the use of molecular biomarkers, specific to pain-generating pathology, can identify the presence of pathological biological processes, even in the apparent absence of anatomical changes. Functional molecular and cellular imaging techniques are currently being investigated, taking advantage of heightened metabolic, hemodynamic, mediator, and cellular changes that are associated with increased nociceptive activity, in order to identify abnormal activity anywhere along the nociceptive pathways in the CNS and peripheral nervous system.

### 4.1. In Vitro Studies

Chronic neuropathic pain is associated with a state of hyperexcitability, resulting from alteration of the excitation threshold, which leads to an increase in action potential. These changes occur due to the increase in the number and activity of voltage pumps dependent on Na and Ca, allowing increased activity of the nerves, as identified by PET studies [156]. The voltage-gated sodium channels Na_v_1.7 and Na_v_1.8 are highly expressed in sensory neurons, and have been identified as promising targets for the development of new analgesics [157]. Immunofluorescence assays and in vitro co-culture models using DRG and dorsal horn (DH) neurons in a three-compartment microfluidic platform showed that while blocking presynaptic Na_v_1.7 and Na_v_1.8 channels is effective in reducing synaptic transmission in uninjured cultures, the same blockers are ineffective in cultures where the DRG axons in the periphery compartment had been axotomized [55]. Live cell imaging and surface channel labelling in primary rat DRG neuron cultures showed that treatment with paclitaxel (PTX), which causes dose-limiting chemotherapy-induced peripheral neuropathy (CIPN), is associated with increased levels of endogenous Na_v_1.7 channels in DRG and long-distance axonal vesicular transport in sensory axons [158]. An immunofluorescence assay with immortalized DRG neuronal cell line (differentiated F11 cell line) identified several potential neuroprotective drugs, such as α-lipoic acid, pregabalin, and melatonin, and confirmed the same effect of felodipine and nitrendipine [159].

Glial activation is a key process in chronic pain states. A study based on immunofluorescence staining that assessed the efficacy of gabapentin in CNP showed that the development and maintenance of hypersensitivity after spinal/peripheral nerve injury is correlated with activation of microglia and astrocytes that present large cell bodies and thick processes. Additionally, glial activation is accompanied by an increase in ionized calcium-binding adapter molecule 1 (Iba-1), as a microglial biomarker; GFAP, as an astrocytic biomarker; the VGCC α2/δ-1 subunit in primary afferent fiber terminals and dorsal horn neurons; and fractalkine/CX3CL1, a putative activator of microglia in the spinal dorsal horn, and its receptor CX3CR1/GPR13, mainly expressed in the spinal microglia [160]. In chronic neuropathic pain, activated microglia and other macrophages express, on the cell surface, a specific protein (TSPO). Imaging techniques can identify and locate activated microglia using radioligands that connect to TSPO, and thus identify the areas with increased neuroinflammation and sensitization [161]. Upregulation of sigma-1 receptors (S1R) occurs in Schwann cells and macrophages at the site of inflammation. Using the 18F-FTC-146, as specific PET radiotracer sigma-1 receptor ligand it was shown that increased uptake in the case of a neuroma caused by nerve injury was correlated with increased receptor expression [162].

The endocannabinoid system, which plays an important part in pain perception, has also been investigated by means of imaging studies in preclinical research. Expressed by peripheral immune cells, neurons, and glia [163], such as microglia and astrocytes, the cannabinoid type 2 receptor (CB2R) was identified as a promising therapeutic target for immunological modulation [164,165], its activation decreasing inflammation [166,167]. CB2 receptors are also upregulated in the CNS and DRG by pathological pain condition [168]. The dorsal spinal cord microglia is known as an important site involved in CB2-receptor-mediated analgesia, while the overexpression of P2Y_12_ and P2Y_13_ purinoceptors in spinal dorsal horn microglia is involved in neuropathic pain [169]. Endocannabinoid anandamide-treated primary cultures of microglial cells showed an alleviation of *E. coli* lipopolysaccharide (LPS)-induced neuroinflammation, an effect primarily controlled by the CB2 receptors [170]. GPR55, a G-protein-coupled receptor highly expressed in large-diameter DRG neurons, has a potential role in the pathophysiology of pain [171]. Immunostaining of mouse DRG showed that it is a cannabinoid receptor that increases intracellular calcium in HEK293 cells and DRG neurons after its activation by cannabinoids, such as Δ^9^-tetrahydrocannabinol (THC) and several endogenous cannabinoids [172], and plays a putative role in modulating nociceptor excitability and neuropathic pain [171].

Chronic pain also induces significant metabolic changes. In the proximity of the primary neuronal lesion, inflammatory mediators are released, which can in turn be identified by modern imaging techniques. Substance P and its receptor, located in the dorsal root ganglion and dorsal horn of the spinal cord (neurokinin-1 receptor), play a major role in the development of hypersensitivity associated with neuropathic pain [161]. The use of radiolabeled neuropeptides and their evaluation via imaging may be used to identify sources of chronic pain receptor mediators. Additionally, inflammation is associated with an increase in metabolism, and thus with increased glucose requirements. Using 18F-fluorodeoxyglucose (18F-FDG), a radiopharmaceutical marker that mimics the role of glucose, we can evaluate via PET its internalization in areas with high metabolism [161]. In a rat model, Behera et al. were able to localize increased 18F-FDG uptake in injured nerves using PET/MRI hybrid imaging. The 18F-FDG uptake correlated well with behavioral measurements of allodynia in the affected paw. In contrast, control nerves in the contralateral normal limb of the subject and in control asymptomatic animals showed significantly less 18F-FDG uptake [173].

### 4.2. In Vivo Preclinical Imaging

Molecular biology has promoted the development of new molecularly targeted diagnostic and therapeutic methods. However, in vitro discoveries cannot always be replicated in vivo. Obviously, the best method of in vivo study of pathophysiological mechanisms would be direct research on human subjects, but this is often impossible due to ethical limitations. Therefore, small animal study models are an important bridge between molecular findings and the implementation of clinically relevant diagnostic techniques or therapies. Studies on small animal models have previously been based on ex vivo tissue sectioning and microscopy. These methods require the existence of a histopathological laboratory, and do not allow the longitudinal study of a single animal. In this sense, out of the need for simplification and modernization, imaging-based methods have become important non-invasive techniques for in vivo investigation of animal research models. Imaging offers the possibility of serial, uniform, automated, non-invasive, and comparable studies on subjects, with advantages and reduction in the number of animals and experimental costs. [174].

As each imaging method has its own advantages and disadvantages, the effective choice of the most appropriate imaging technique or techniques should be made according to the hypotheses and questions whose answers are to be discovered in the published research (Table 3) [175,176].

Neuropathic pain is associated in the brain with both functional changes (such as changes in neuronal excitability) and structural changes (including the increased density of dendrites and presynaptic axon boutons). Although there are specific peculiarities of each brain area, modulation of neuronal plasticity has a significant therapeutic potential in this type of pathology [177]. In a model of nerve-injury-induced neuropathic pain, using PET techniques and specific radiotracers, mGluR5 upregulation was shown in brain areas such as the prelimbic region and medial prefrontal cortex [178]. The antidepressive effect of mGluR5 antagonists (also confirmed by other studies) has an even higher significance since neuropathic pain is often associated with changes in mental status. One explanation could be that chronic pain produced by injuries of peripheric nerves is linked with alterations of brain structures involved in emotional processing [178,179]. Similarly, in the primary somatosensory cortex, upregulation of these receptors was associated with the astrocytic release of TSP-1 (synaptogenic thrombospondin 1) and elevated dendritic spine turnover in the pyramidal neurons [177]. Administration of specific antagonists reduced tactile hypersensitivity and depressive-like behavior in a forced swimming test paradigm [178].

One of the most promising applications of imaging studies in chronic pain comes from the ability to screen for the effectiveness of a new analgesic drug. Imagistic screening of drugs represents a predictive tool that allows the selection of new compounds that act on limbic structures and sensorimotor system, important areas for neuropathic pain pathways. For example, using pharmacological MRI (phMRI), Hooker et al. (2014) [180] showed that gabapentin significantly altered the brain’s functional circuits when administered in the treatment of neuropathic pain. Variations of receptors expression, also identifiable using PET analysis, can explain the differences in responses to treatment between patients and also the depression-like behavior/emotional disturbances associated with neuropathic pain [179]. Of note, by means of fMRI studies, we now know that drugs exhibit specificity regarding the activation of different brain areas [179].

A promising class of compounds for the treatment of neuropathic pain are the S1R antagonists, receptors that were originally thought to be part of the opioid receptor family. Administration of a non-radioactive [19F]FTC-146 radioligand and an S1R antagonist in an animal model of neuropathic pain reduced allodynia and equalized the von Frey test response between the injured and healthy paw. These data correlate with the fact that at the site of subacute peripheral nerve injury, there is elevated S1R expression and increased uptake of 18FTC-146 at PET investigation that parallels the intensity of pain [181]. In addition, S1R modulates the activity of ion channels (such as calcium and potassium channels) whose dysfunctions play a role in the generation of pain. Moreover, S1R stimulation amplifies NMDA-receptor-induced pain and plays a role in mechanical and thermal hypersensitivity [181].

Because CNP is such a complex condition with multiple activating pathways, multifunctional compounds acting simultaneously on multiple receptors or multiple pathways have emerged as an attractive alternative. Several drug combinations, such as fused opioid agonist/neurokinin 1 antagonist peptidomimetics [182], bifunctional opioid/melanocortin-4 receptor peptidomimetics [183], or dual enkephalinase inhibitors [184] are currently being investigated. These combinations of drugs have the advantage of exhibiting synergic pharmacodynamic effects with fewer side effects and requiring just one regulatory approval [185]. The effectiveness of multitargeted drugs is directly proportional with the capacity of acting on several stages of the disease’s neuroprogression and its ″phenotypic penetrance″. It is important to modulate both neuronal neuroplasticity and neurochemical immunological factors that contribute to the establishment of vicious brain circuits that allow self-maintenance of pain and related behavioral changes [186].

Another avenue of innovation in chronic neuropathy pharmacotherapy is the identification of new modalities and new compounds able to cross the brain–blood barrier and deliver targeted drug molecules. An example of this is colloidal nanocarriers (nanocrystals, polymeric nanoparticles, nanocomposite conjugated inorganic nanoparticles) that can carry drugs, peptides, and nucleic acids [187,188]. Exostomes are another potential new treatment approach. As nanosized (30–120 nm) membrane vesicles, exosomes easily cross the brain–blood barrier and transfer cargo to brain cells, such as neurons, microglia, oligodendrocytes [189,190]. Current data indicate the possibility of using 64 CuCl2 ligands for MRI identification of the pharmacokinetic characteristics of these small extracellular vesicles in the brain [190,191]. In the C57bl/6 mouse model of stroke, Perets and colleagues (2019) showed that mesenchymal stem cell (MSC)-derived exosomes labeled with glucose-coated gold nanoparticles and administered intranasally specifically accumulated in areas of brain injury [192,193]. Either by active methods or by passive encapsulation, these exostomes can deliver therapeutic chemicals or RNAs to brain areas. In a rodent model of acute brain inflammation, catalase-loaded exosomes exhibited neuroprotective effects and decreased microgliosis and astrocytosis [189]. So far, targeting the delivery of exosomes to the brain can be achieved using extracellular vesicles derived from neural stem cells, exosomes derived from macrophages expressing LFA-1 and C-type lectin receptors, and exosomes incorporating a polypeptide fragment of rabies virus glycoprotein [190].

Taking into consideration the fact that neural plasticity in specific brain areas is linked to the genesis of neuropathic pain, we can only speculate that substances that influence brain neuroplasticity will also have an effect in modulating neuropathic pain. Modulating long-term potentiation processes in the anterior cingulate cortex, substances such as tetrodotoxin (TTX), CNQX—a competitive α-amino-3-hydroxy-5-methyl-4-isoxazolepropionic acid (AMPA) receptor antagonist, z-pseudosubstrate inhibitory peptide (ZIP), anisomycin, rapamycin, and PCC0208009—an indolamine 2,3-dioxygenase 1 inhibitor, were shown to alleviate allodynia in neuropathic pain [177].

In the future, the identification of new molecules effective in the treatment of neuropathy will require a paradigm shift. One way to increase the efficacy of these compounds is to use targeted therapy depending on disease subtypes (characterized by the same pathophysiological mechanism). As Baron (2017) [194] also states, based on the type of fibers affected, the mechanism of nerve injury/lesion, and associated symptoms, several clusters of peripheral neuropathy can be defined. This type of stratification of patient groups allows the development of specific therapeutic protocols [195].

**Figure 2:**
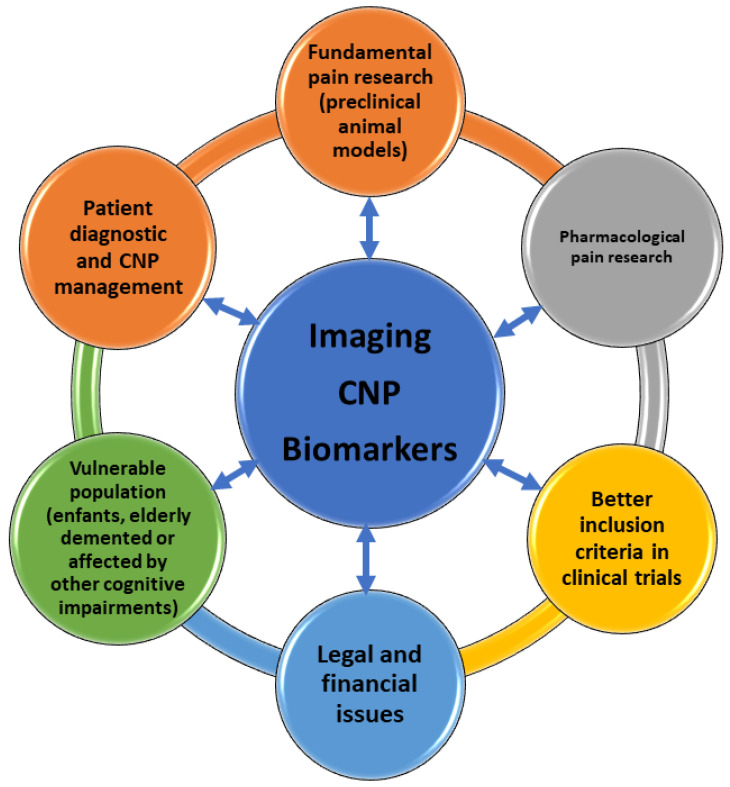
Pain imaging biomarker development implications.

Another criterion to select clusters is the use of data on channel-specific dysfunction. We predict that in the future new therapeutic agents with analgesic effects will be identified based on correlations between genetic mutations in ion channels and changes in pain sensations. This category may include substances that act on Nav1.7 voltage-gated sodium channels, TRPV1 cation channels, N-type calcium channels, AT2 receptors, calcitonin-gene-related peptide receptors, or the NGF–TrkA signaling pathway [185]. Nonetheless, imaging-based drug screening will probably become more and more popular within the research community due to its non-invasive character and the validity of its results (Figure 2).

## 5. Conclusions

Chronic neuropathic pain is a very important public health issue with profound negative implications in many aspects of patients’ individual lives, as well as society, health systems, productivity, and macroeconomics. Therefore, it is imperative to explore new tests to assess this condition, using scientific and technological progress. The development of imaging, especially molecular and functional imaging, provides objectivity and makes the connection between structural changes, receptors involved in the mechanisms of action, and potentially therapeutic or diagnostic molecules by highlighting the place of action and the involved systems. The approval of composite biomarkers, including serological, genetic, clinical, and imaging markers, with high sensitivity and specificity will accelerate and improve diagnosis, staging, predictive and prognostic evaluation, stratification (phenotyping) and inclusion in trials, and the development of therapeutic options (pharmacological, biomedical) through preclinical, translational, clinical studies. This review covers a useful approach for the identification of new promising reliable and quantifiable imaging biomarkers that could impact the management of CNP. Thus, there is an urgent need to strengthen development by setting up specialized study groups that bring together individual research projects and test hypotheses through complex combined studies of molecular biology, pharmacology, and advanced imaging.

## Figures and Tables

**Table 1:** A summary of the neuroimaging-based biomarkers in chronic neuropathic pain.

Structural Changes in Gray Matter Volume, Cortical Thickness, Integrity and Connectivity of White Matter		
Neuroimaging Method	Stimuli	Findings
MRI	Neuropathic back pain patients [77,92,93,94]	Altered whole-brain gray matter volume and reduction in the gray matter density in dorsolateral PFC and thalamus.
MRI DTI	Chronic complex regional pain syndrome [95]	Gray matter atrophy in the right medial PFC and anterior insula and localized reduced white matter anisotropy.
fMRI	Carpal tunnel syndrome [96]	Gray matter volume was decreased in the SI, thalamus, and frontal areas
fMRI MRI	Trigeminal neuropathic pain [97]	Gray matter cortical thickening in the SI and medial and posterior insula; cortical thinning in ACC, anterior insula, PFC, and frontal pole.
fMRI MRI	Trigeminal neuropathic pain [98]	Increased volume of gray matter in the thalamus, basal ganglia, amygdala, and PAG. Increased cortical thickness in the contralateral SI and SII and frontal pole; thinner cortex in the ACC, insula, and the orbitofrontal cortex.
fMRI MRI	Trigeminal neuropathic pain [99]	Reduced volume of gray matter in the SI, anterior insula, putamen, nucleus accumbens, and thalamus. Increased gray matter volume in posterior insula.
DTI	Trigeminal neuropathic pain [100]	Reduced fractional anisotropy of gray matter and increased radial diffusivity.
**Functional alterations in regional cerebral blood flow (rCBF) and** **resting-state functional connectivity (rsFC)**		
**Neuroimaging method**	**Stimuli**	**Findings**
PET	Neuropathic pain patients versus normal controls [101]	Thalamic CBF reduction.
PET	Neuropathic pain patients versus relief [102]	Increased CBF in hypothalamus, PAG, PFC, anterior ACC, insula, PPC, and cerebellum. CBF reduction was noted in the primary auditory cortex.
PET	Mononeuropathy [103]	Increased CBF in the anterior insula, SII, thalamus, SI, parietal area, midbrain, and cerebellum; meanwhile, its reduction was reported in rostral ACC.
fMRI	Diabetic neuropathic pain [104]	Decreased rsFC between cortex and thalamus.
fMRI	Chronic back pain [105]	Increased rsFC between default mode network and perigenual anterior ACC, insula, and inferior parietal lobe.
fMRI	Chronic back pain [106]	Increased rCBF in SI, SII, PFC, and IC.
PET	Neuropathic pain after spinal cord injury [107]	Increased rCBF in ACC, thalamus, PFC, and parietal cortex after spinal cord stimulation.
**Neurochemical changes**		
PET	Trigeminal neuropathic pain [108]	Reduced opioid receptor binding in the left nucleus accumbens.
PET	Trigeminal neuropathic pain [109]	Increased opioid receptor binding in PFC, IC, ACC, PPC, thalamus, and basal ganglia.
PET	Complex regional pain syndrome [110]	Decreased opioid receptor binding in hippocampus and amygdala, and increased in PFC.
H-MRS	Neuropathic pain individuals [111]	Reduction in N-acetylaspartate concentrations in contralateral thalamus.
H-MRS	Chronic back pain [112]	N-acetylaspartate and glucose level reduction.
H-MRS	Complex regional pain syndrome [113]	N-acetylaspartate concentration reduction in dorsolateral PFC.
H-MRS	Neuropathic pain following spinal cord injury [114]	Reduced N-acetylaspartate levels in thalamus.
H-MRS	Diabetic neuropathy [115]	Reduced N-acetylaspartate and creatine levels in dorsolateral PFC and thalamus.
H-MRS	Neuropathic pain after nerve injury [116]	Elevated glutamate levels and low GABA concentrations in thalamus.
MRI fMRI H-MRS	Neuropathic pain after spinal cord injury [99]	Reduction in N-acetylaspartate level and GABA content in thalamus.
H-MRS	Chronic back pain [117]	Strong variance of glutamate/GABA ratios in IC and ACC.
H-MRS	Chronic back pain [118]	Lower N-acetylaspartate levels and higher glutamate–glutamine/creatine and glutamate–glutamine/myoinositol ratios in ACC.
MRI H-MRS	Neuropathic pain after spinal cord injury [119]	Higher levels of myoinositol, choline, and creatine; lower levels of N-acetyl aspartate/myoinositol and glutamate–glutamine/myoinositol in ACC.

MRI, magnetic resonance imaging; PET, positron emission tomography; IC, insular cortex; SI and SII, primary and secondary somatosensory cortices; ACC, anterior cingulate cortex; PFC, prefrontal cortex; fMRI, functional magnetic resonance imaging; PAG, periaqueductal gray; PPC, posterior parietal cortex; DTI, diffusion tensor imaging; H-MRS, proton magnetic resonance spectroscopy; GABA, γ-aminobutyric acid; rsFC, resting-state functional connectivity.

**Table 2:** Imaging changes/potential imaging biomarkers in response to various neurostimulation methods for chronic pain.

DBS Implant		
Area of Interest	Imaging Method	Imaging Changes
Periaqueductal gray	[11C]diprenorphine (DPN)—PET	DBS activation caused a focal reduction in [11C]DPN VT in the PAG, consistent with stimulation-evoked release of endogenous opioids [142].
Thalamus	[15O]H2O PET	Changes in rCBF during thalamic DBS revealed stimulation-related increases within the ACC, the globus pallidus, and a region lateral to the thalamus identified as the internal capsule [143].
VS/ALIC	fMRI	In response to pain, patients in the DBS OFF state showed significant activation in the same regions as healthy controls (thalamus, insula, and operculum) and in additional regions (orbitofrontal and superior convexity cortical areas). DBS significantly reduced activation of these additional regions and introduced foci of significant inhibitory activation (*p* < 0.001) in the hippocampi when painful stimulation was applied to the affected side [144].
Non-specific	MRI DTI	Patients for whom the DBS electrodes were within the DTI targets experienced better outcomes than those for whom the electrodes were not [145].
VPL and PVG areas	SPECT	DBS consistently increased perfusion in the posterior subcortical region between VPL and PVG, regardless of the site of stimulation. Thalamic and dual-target DBS increased thalamic perfusion, yet PVG DBS decreased perfusion in the PVG-containing midbrain region and thalamus. Dual-target stimulation decreased anterior cingulate and insular cortex perfusion [146].
ACC	MEG	Long-term functional brain changes as a result of continuous DBS over one year led to specific changes in the activity in the dissociable regions of caudal and rostral ACC [147].
**SCS implant**		
**Imaging method**	**Imaging changes**	
fMRI, PET, SPECT, H-MRS	Increased activity in frontal regions of the cortex, as well as identifying the ACC and thalamus as mediators of the pain experience and potentially key components determining the influence of SCS at supraspinal levels. Prior to stimulation, poor responders to SCS showed increased thalamic activation, whereas good responders showed almost no activation in the thalamus [148].	
[15O]H2O PET	Comparison of rCBF before and after SCS showed significant rCBF increases in the right thalamus, right orbitofrontal cortex (BA11), left inferior parietal lobule (BA7), right superior parietal lobule (BA7), left anterior cingulate cortex (ACC) (BA24), and left lateral prefrontal cortex (BA10) [107].	
[18F]FDG-PET	Burst stimulation modulated the dorsal ACC (i.e., medial pain pathway) more than tonic stimulation [149].	
**DRG stimulation**		
**Imaging method**	**Imaging changes**	
fMRI BOLD	During noxious paw stimulation, ganglionic field stimulation (GFS) attenuates the BOLD signal in brain regions composing the ascending neospinothalamic system, specifically the contralateral thalamic VPL/VPM nuclei and cortical S1 and S2. This ascending sensory pathway subserves the sensory–discriminative dimension of pain, composed of immediate pain awareness and spatial attentiveness to painful stimuli [150].	
**MCS implant**		
**Imaging method**	**Imaging changes**	
[15O]H2O PET	Compared to baseline, turning on the stimulator was associated with CBF increase in the contralateral (anterior) midcingulate cortex (aMCC, BA24 and 32) and in the dorsolateral prefrontal (BA10) cortex. The most important changes in CBF were observed in the 75 min after discontinuation of MCS (OFF). This poststimulation period was associated with CBF increases in a large set of cortical and subcortical regions (from posterior MCC (pMCC) to pregenual (pg) ACC, orbitofrontal cortex, putamen, thalami, posterior cingulate, and prefrontal areas, and in the brainstem. CBF changes in the poststimulation period correlated with pain relief [151].	
[18F]FDG micro-PET (mPET)	Changes in brain activity were observed in the striatum, thalamic area, and cerebellum [152].	
**TENS**		
**Area of interest**	**Imaging method**	**Imaging changes**
Carpal tunnel syndrome	fMRI	Within 0 to 25 minutes after TENS treatment, significant fMRI signal decrease for digit 2 was observed in the secondary somatosensory regions, ipsilateral primary motor cortex (M1), contralateral supplementary motor cortex (SMA), contralateral parahippocampal gyrus, contralateral lingual gyrus, and bilateral superior temporal gyrus. Within 30 to 35 minutes after TENS treatment, a significant fMRI signal decrease for digit 3 was detected in the contralateral M1 and contralateral SMA only in the TENS group [153].
**rTMS**		
**Area of interest**	**Imaging method**	**Imaging changes**
Primary motor cortex	MRI DTI	The rTMS-effective group had a higher delineation ratio of the corticospinal tract (CST) (*p* = 0.02) and the thalamocortical tract (TCT) (*p* = 0.005) than the rTMS-ineffective group. Results suggest that the TCT also plays a role in pain reduction via rTMS of the primary motor cortex, and that the efficacy of rTMS is predictable by fiber tracking [154].

DBS, deep brain stimulation; MCS, motor cortex stimulation; SCS, spinal cord stimulation; DRG, dorsal root ganglion; TENS, transcutaneous electrical nerve stimulation; rTMS, repetitive transcranial magnetic stimulation; VS/ALIC, ventral striatum/anterior limb of the internal capsule; VPL, ventroposterolateral thalamic nucleus; VPM, ventral posteromedial nucleus; PVG, periventricular gray area; ACC, anterior cingulate cortex; VT, ventral tuberal nucleus; DPN, [11C]diprenorphine; PET, positron emission tomography; mPET, micropositron emission tomography; FDG, fluorodeoxyglucose; fMRI, functional magnetic resonance imaging; MEG, magnetoencephalography; MRI, magnetic resonance imaging; DTI, diffusion tensor imaging; BOLD, blood-oxygenation-level-dependent; SPECT, single-photon emission computer tomography; H-MRS, proton magnetic resonance spectroscopy; rCBF, regional cerebral blood flow; GABA, gamma-aminobutyric acid; GFS, ganglionic field stimulation; aMCC/pMCC, anterior/posterior midcingulate cortex; SMA, supplementary motor cortex; CST, corticospinal tract; TCT, thalamocortical tract.

**Table 3:** Approaches to key questions on drug development using appropriate preclinical imaging methods for translational research (adapted after [176]).

Issue	Question	Preclinical Imaging Approach
Validation	Does the therapeutic target play a key role in the pathophysiological mechanism?	MRI whole-body scan
Biodistribution	Does the molecule reach the targeted tissue? Can pharmacologically active concentrations be reached for the compound being studied?	Radiolabeling of the compound to study the distribution or interactions in the target area (use of PET, SPECT, or MRS)
Interactions	Does the studied molecule interact with the target receptor? What is the relationship between dose and effect?	Saturation studies (plasma concentration–occupancy–time) using PET or SPECT radiolabeling
Pharmacodynamics	What is the effect and duration of the compound?	Dynamic biochemical studies, structural or functional studies (non-ionizing radiation imaging techniques are recommended for long-term effects follow-up)
Toxicology	Does the compound have acute or chronic toxic effects?	Dynamic biochemical studies, structural or functional studies of at risk organs (MRI, CT, US)

MRI, magnetic resonance imaging; PET, positron emission tomography; H-MRS, proton magnetic resonance spectroscopy; SPECT, single-photon emission computed tomography; CT, computed tomography; US, ultrasonography.

## Data Availability

No new data were created or analyzed in this study. Data sharing is not applicable to this article.

## References

[1] Pearce J.M.S. (2013). The Neurology of Aretaeus: Radix Pedis Neurologia. Eur. Neurol..

[2] Heydari M., Shams M., Hashem Hashempur M., Zargaran A., Dalfardi B., Borhani-Haghighi A. (2015). The origin of the concept of neuropathic pain in early medieval persia (9 TH-12 TH CENTURY CE) izvorište pojma neurogene boli u ranosrednjevjekovnoj perziji (9.-12. STOLJEĆE). Acta Med.-Hist. Adriat..

[3] Scholz J., Finnerup N.B., Attal N., Aziz Q., Baron R., Bennett M.I., Benoliel R., Cohen M., Cruccu G., Davis K.D. (2019). The IASP Classification of Chronic Pain for ICD-11: Chronic Neuropathic Pain. Pain.

[4] VanDenKerkhof E.G., Mann E.G., Torrance N., Smith B.H., Johnson A., Gilron I. (2016). An Epidemiological Study of Neuropathic Pain Symptoms in Canadian Adults. Pain Res. Manag..

[5] Torrance N., Smith B.H., Bennett M.I., Lee A.J. (2006). The Epidemiology of Chronic Pain of Predominantly Neuropathic Origin. Results From a General Population Survey. J. Pain..

[6] Torrance N., Ferguson J.A., Afolabi E., Bennett M.I., Serpell M.G., Dunn K.M., Smith B.H. (2013). Neuropathic Pain in the Community: More under-Treated than Refractory?. Pain.

[7] Ohayon M.M., Stingl J.C. (2012). Prevalence and Comorbidity of Chronic Pain in the German General Population. J. Psychiatr. Res..

[8] Bouhassira D., Lantéri-Minet M., Attal N., Laurent B., Touboul C. (2008). Prevalence of Chronic Pain with Neuropathic Characteristics in the General Population. Pain.

[9] Harifi G., Amine M., Ait Ouazar M., Boujemaoui A., Ouilki I., Rekkab I., Belkhou A., el Bouchti I., Niamane R., el Hassani S. (2013). Prevalence of Chronic Pain with Neuropathic Characteristics in the Moroccan General Population: A National Survey. Pain Med..

[10] Inoue S., Taguchi T., Yamashita T., Nakamura M., Ushida T. (2017). The Prevalence and Impact of Chronic Neuropathic Pain on Daily and Social Life: A Nationwide Study in a Japanese Population. Eur. J. Pain.

[11] Yawn B.P., Wollan P.C., Weingarten T.N., Watson J.C., Hooten W.M., Melton L.J. (2009). The Prevalence of Neuropathic Pain: Clinical Evaluation Compared with Screening Tools in a Community Population. Pain Med..

[12] de Moraes Vieira É.B., Garcia J.B.S., da Silva A.A.M., Mualem Araújo R.L.T., Jansen R.C.S. (2012). Prevalence, Characteristics, and Factors Associated With Chronic Pain With and Without Neuropathic Characteristics in São Luís, Brazil. J. Pain Symptom Manag..

[13] Doth A.H., Hansson P.T., Jensen M.P., Taylor R.S. (2010). The Burden of Neuropathic Pain: A Systematic Review and Meta-Analysis of Health Utilities. Pain.

[14] Bennett M.I., Smith B.H., Torrance N., Potter J. (2005). The S-LANSS Score for Identifying Pain of Predominantly Neuropathic Origin: Validation for Use in Clinical and Postal Research. J. Pain.

[15] Jensen M.P., Chodroff M.J., Dworkin R.H. (2007). The Impact of Neuropathic Pain on Health-Related Quality of Life: Review and Implications. Neurology.

[16] Phillips C.J. (2009). The Cost and Burden of Chronic Pain. Rev Pain.

[17] Berger A., Sadosky A., Dukes E., Edelsberg J., Oster G. (2012). Clinical Characteristics and Patterns of Healthcare Utilization in Patients with Painful Neuropathic Disorders in UK General Practice: A Retrospective Cohort Study. BMC Neurol..

[18] Schaefer C., Sadosky A., Mann R., Daniel S., Parsons B., Tuchman M., Anschel A., Stacey B.R., Nalamachu S., Nieshoff E. (2014). Pain Severity and the Economic Burden of Neuropathic Pain in the United States: BEAT Neuropathic Pain Observational Study. Clinicoecon. Outcomes Res..

[19] Lundberg D., Axelsson S., Boivie J., Eckerlund I., Gerdle B., Gullacksen A.-C., Breivik H., Bunch E.H., Hasselström J., Linton S.J. (2006). Summary and Conclusions of the SBU Report on: Methods of Treating Chronic Pain A Systematic Review Scientific Reviewers: English Translation.

[20] Lachaine J., Gordon A., Choinière M., Collet J., Dion D., Tarride J.-E. (2007). Painful Neuropathic Disorders: An Analysis of the RéGie De L’Assurance Maladie Du QuéBec Database. Pain Res. Manag..

[21] Von Hehn C.A., Baron R., Woolf C.J. (2012). Deconstructing the Neuropathic Pain Phenotype to Reveal Neural Mechanisms. Neuron.

[22] Finnerup N.B., Otto M., McQuay H.J., Jensen T.S., Sindrup S.H. (2005). Algorithm for Neuropathic Pain Treatment: An Evidence Based Proposal. Pain.

[23] Dworkin R.H., O’Connor A.B., Backonja M., Farrar J.T., Finnerup N.B., Jensen T.S., Kalso E.A., Loeser J.D., Miaskowski C., Nurmikko T.J. (2007). Pharmacologic Management of Neuropathic Pain: Evidence-Based Recommendations. Pain.

[24] Attal N., Cruccu G., Baron R., Haanpää M., Hansson P., Jensen T.S., Nurmikko T. (2010). EFNS Guidelines on the Pharmacological Treatment of Neuropathic Pain: 2010 Revision. Eur. J. Neurol..

[25] Moore A., Derry S., Eccleston C., Kalso E. (2013). Expect Analgesic Failure; Pursue Analgesic Success. BMJ.

[26] Breivik H., Eisenberg E., O’Brien T. (2013). The Individual and Societal Burden of Chronic Pain in Europe: The Case for Strategic Prioritisation and Action to Improve Knowledge and Availability of Appropriate Care. BMC Public Health.

[27] Bouhassira D., Attal N., Alchaar H., Boureau F., Brochet B., Bruxelle J., Cunin G., Fermanian J., Ginies P., Grun-Overdyking A. (2005). Comparison of Pain Syndromes Associated with Nervous or Somatic Lesions and Development of a New Neuropathic Pain Diagnostic Questionnaire (DN4). Pain.

[28] Haussleiter I.S., Richter H., Scherens A., Schwenkreis P., Tegenthoff M., Maier C. (2008). NeuroQuick—A Novel Bedside Test for Small Fiber Neuropathy?. Eur. J. Pain.

[29] Gray P. (2008). Acute Neuropathic Pain: Diagnosis and Treatment. Curr. Opin Anaesthesiol..

[30] Corbett A., Husebo B., Malcangio M., Staniland A., Cohen-Mansfield J., Aarsland D., Ballard C. (2012). Assessment and Treatment of Pain in People with Dementia. Nat. Rev. Neurol..

[31] Rudin M. (2009). Noninvasive Structural, Functional, and Molecular Imaging in Drug Development. Curr. Opin Chem. Biol..

[32] Davis K.D., Aghaeepour N., Ahn A.H., Angst M.S., Borsook D., Brenton A., Burczynski M.E., Crean C., Edwards R., Gaudilliere B. (2020). Discovery and Validation of Biomarkers to Aid the Development of Safe and Effective Pain Therapeutics: Challenges and Opportunities. Nat. Rev. Neurol..

[33] Jensen T.S., Baron R., Haanpää M., Kalso E., Loeser J.D., Rice A.S.C., Treede R.D. (2011). A New Definition of Neuropathic Pain. Pain.

[34] The Pathophysiology of Neuropathic Pain. https://www.practicalpainmanagement.com/pain/neuropathic/pathophysiology-neuropathic-pain.

[35] Woolf C.J., Mannion R.J. (1999). Neuropathic Pain: Aetiology, Symptoms, Mechanisms, and Management. Lancet.

[36] Colloca L., Ludman T., Bouhassira D., Baron R., Dickenson A.H., Yarnitsky D., Freeman R., Truini A., Attal N., Finnerup N.B. (2017). Neuropathic Pain. Nat. Rev. Dis. Primers.

[37] Baron R. (2006). Mechanisms of Disease: Neuropathic Pain--a Clinical Perspective. Nat. Clin. Pract. Neurol..

[38] Seifert F., Maihöfner C. (2009). Central Mechanisms of Experimental and Chronic Neuropathic Pain: Findings from Functional Imaging Studies. Cell. Mol. Life Sci..

[39] Iannetti G.D., Mouraux A. (2010). From the Neuromatrix to the Pain Matrix (and Back). Exp. Brain Res..

[40] Salomons T.v., Iannetti G.D., Liang M., Wood J.N. (2016). The “Pain Matrix” in Pain-Free Individuals. JAMA Neurol..

[41] Moisset X., Bouhassira D. (2007). Brain Imaging of Neuropathic Pain. Neuroimage.

[42] Garcia-Larrea L., Peyron R. (2013). Pain Matrices and Neuropathic Pain Matrices: A Review. Pain.

[43] Kupers R., Lonsdale M.N., Aasvang E., Kehlet H. (2011). A Positron Emission Tomography Study of Wind-up Pain in Chronic Postherniotomy Pain. Eur. J. Pain.

[44] Fu Y., Han J., Ishola T., Scerbo M., Adwanikar H., Ramsey C., Neugebauer V. (2008). PKA and ERK, but Not PKC, in the Amygdala Contribute to Pain-Related Synaptic Plasticity and Behavior. Mol. Pain.

[45] Pedersen L.H., Scheel-Krüger J., Blackburn-Munro G. (2007). Amygdala GABA-A Receptor Involvement in Mediating Sensory-Discriminative and Affective-Motivational Pain Responses in a Rat Model of Peripheral Nerve Injury. Pain.

[46] Tracey I., Mantyh P.W. (2007). The Cerebral Signature for Pain Perception and Its Modulation. Neuron.

[47] Elbert T., Flor H., Birbaumer N., Knecht S., Hampson S., Larbig W., Taub E. (1994). Extensive Reorganization of the Somatosensory Cortex in Adult Humans after Nervous System Injury. Neuroreport.

[48] Nikolajsen L., Ilkjær S., Krøner K., Christensen J.H., Jensen T.S. (1997). The Influence of Preamputation Pain on Postamputation Stump and Phantom Pain. Pain.

[49] Draganski B., Moser T., Lummel N., Gänssbauer S., Bogdahn U., Haas F., May A. (2006). Decrease of Thalamic Gray Matter Following Limb Amputation. Neuroimage.

[50] Raichle M.E. (2015). The Brain’s Default Mode Network. Annu. Rev. Neurosci..

[51] Baliki M.N., Geha P.Y., Apkarian A.V., Chialvo D.R. (2008). Beyond Feeling: Chronic Pain Hurts the Brain, Disrupting the Default-Mode Network Dynamics. J. Neurosci..

[52] Scholz J., Woolf C.J. (2007). The Neuropathic Pain Triad: Neurons, Immune Cells and Glia. Nat. Neurosci..

[53] Tsuda M., Beggs S., Salter M.W., Inoue K. (2013). Microglia and Intractable Chronic Pain. Glia.

[54] Haberberger R.V., Barry C., Matusica D. (2020). Immortalized Dorsal Root Ganglion Neuron Cell Lines. Front. Cell. Neurosci..

[55] Vysokov N., McMahon S.B., Raouf R. (2019). The Role of Na V Channels in Synaptic Transmission after Axotomy in a Microfluidic Culture Platform. Sci. Rep..

[56] Shin S.M., Itson-Zoske B., Cai Y., Qiu C., Pan B., Stucky C.L., Hogan Q.H., Yu H. (2020). Satellite Glial Cells in Sensory Ganglia Express Functional Transient Receptor Potential Ankyrin 1 That Is Sensitized in Neuropathic and Inflammatory Pain. Mol. Pain.

[57] Haberberger R.V., Barry C., Dominguez N., Matusica D. (2019). Human Dorsal Root Ganglia. Front. Cell. Neurosci..

[58] Machelska H., Celik M. (2016). Recent Advances in Understanding Neuropathic Pain: Glia, Sex Differences, and Epigenetics. F1000Res.

[59] Deumens R., Jaken R.J.P., Knaepen L., van der Meulen I., Joosten E.A.J. (2009). Inverse Relation between Intensity of GFAP Expression in the Substantia Gelatinosa and Degree of Chronic Mechanical Allodynia. Neurosci. Lett..

[60] Gwak Y.S., Hulsebosch C.E., Leem J.W. (2017). Neuronal-Glial Interactions Maintain Chronic Neuropathic Pain after Spinal Cord Injury. Neural Plast..

[61] Wang X., Tian S., Wang H., Liu P., Zheng H., Wu L., Liu Q., Wu W. (2020). Botulinum Toxin Type A Alleviates Neuropathic Pain and Suppresses Inflammatory Cytokines Release from Microglia by Targeting TLR2/MyD88 and SNAP23. Cell Biosci..

[62] Zhang L., Xie R., Yang J., Zhao Y., Qi C., Bian G., Wang M., Shan J., Wang C., Wang D. (2019). Chronic Pain Induces Nociceptive Neurogenesis in Dorsal Root Ganglia from Sox2-Positive Satellite Cells. Glia.

[63] Wang H., Sun H., della Penna K., Benz R.J., Xu J., Gerhold D.L., Holder D.J., Koblan K.S. (2002). Chronic Neuropathic Pain Is Accompanied by Global Changes in Gene Expression and Shares Pathobiology with Neurodegenerative Diseases. Neuroscience.

[64] Yu X., Basbaum A., Guan Z. (2021). Contribution of Colony-Stimulating Factor 1 to Neuropathic Pain. Pain Rep..

[65] Chen G., Zhang Y.Q., Qadri Y.J., Serhan C.N., Ji R.R. (2018). Microglia in Pain: Detrimental and Protective Roles in Pathogenesis and Resolution of Pain. Neuron.

[66] Prabakaran S. (2016). CSF-1 Delivers a Painful Signal. Sci. Signal..

[67] Lindå H., Sköld M.K., Ochsmann T. (2011). Activating Transcription Factor 3, a Useful Marker for Regenerative Response after Nerve Root Injury. Front. Neurol..

[68] Guan Z., Kuhn J.A., Wang X., Colquitt B., Solorzano C., Vaman S., Guan A.K., Evans-Reinsch Z., Braz J., Devor M. (2016). Injured Sensory Neuron-Derived CSF1 Induces Microglial Proliferation and DAP12-Dependent Pain. Nat. Neurosci..

[69] Chattopadhyay S., Myers R.R., Janes J., Shubayev V. (2007). Cytokine Regulation of MMP-9 in Peripheral Glia: Implications for Pathological Processes and Pain in Injured Nerve. Brain Behav. Immun..

[70] Gu H.W., Xing F., Jiang M.J., Wang Y., Bai L., Zhang J., Li T.T., Zhang W., Xu J.T. (2019). Upregulation of Matrix Metalloproteinase-9/2 in the Wounded Tissue, Dorsal Root Ganglia, and Spinal Cord Is Involved in the Development of Postoperative Pain. Brain Res..

[71] Tauber S., Scheider-Stock R., Ullrich O. (2009). Investigating Immunmodulatory Mechanisms of Cannabinoids: The Role of MMP-9. Cell Commun. Signal..

[72] Kawasaki Y., Xu Z.Z., Wang X., Park J.Y., Zhuang Z.Y., Tan P.H., Gao Y.J., Roy K., Corfas G., Lo E.H. (2008). Distinct Roles of Matrix Metalloproteases in the Early- and Late-Phase Development of Neuropathic Pain. Nat. Med..

[73] Calvo M., Zhu N., Grist J., Ma Z., Loeb J.A., Bennett D.L.H. (2011). Following Nerve Injury Neuregulin-1 Drives Microglial Proliferation and Neuropathic Pain via the MEK/ERK Pathway. Glia.

[74] Martucci K.T., Mackey S.C. (2016). Imaging Pain. Anesthesiol. Clin..

[75] Davis K.D. (2011). Neuroimaging of Pain: What Does It Tell Us?. Curr. Opin. Support. Palliat. Care.

[76] Zhang Z., Gewandter J.S., Geha P. (2022). Brain Imaging Biomarkers for Chronic Pain. Front. Neurol..

[77] Ung H., Brown J.E., Johnson K.A., Younger J., Hush J., Mackey S. (2014). Multivariate Classification of Structural MRI Data Detects Chronic Low Back Pain. Cereb. Cortex.

[78] Logothetis N.K. (2008). What We Can Do and What We Cannot Do with FMRI. Nature.

[79] Alomar S., Bakhaidar M. (2018). Neuroimaging of Neuropathic Pain: Review of Current Status and Future Directions. Neurosurg. Rev..

[80] Baliki M.N., Chialvo D.R., Geha P.Y., Levy R.M., Harden R.N., Parrish T.B., Apkarian A.V. (2006). Chronic Pain and the Emotional Brain: Specific Brain Activity Associated with Spontaneous Fluctuations of Intensity of Chronic Back Pain. J. Neurosci..

[81] Seymour B., Mano H., Kotecha G., Leibnitz K., Matsubara T., Sprenger C., Nakae A., Shenker N., Shibata M., Voon V. (2018). Classification and Characterisation of Brain Network Changes in Chronic Back Pain: A Multicenter Study. Wellcome Open Res..

[82] Chang L., Munsaka S.M., Kraft-Terry S., Ernst T. (2013). Magnetic Resonance Spectroscopy to Assess NeuroInflammation and Neuropathic Pain. J. Neuroimmune Pharmacol..

[83] (2011). What Is an MRI Scan and What Can It Do?. Drug Ther. Bull..

[84] Ghanouni P., Behera D., Xie J., Chen X., Moseley M., Biswal S. (2012). In Vivo USPIO Magnetic Resonance Imaging Shows That Minocycline Mitigates Macrophage Recruitment to a Peripheral Nerve Injury. Mol. Pain.

[85] Lu F.-M., Yuan Z. (2015). PET/SPECT Molecular Imaging in Clinical Neuroscience: Recent Advances in the Investigation of CNS Diseases. Quant. Imaging Med. Surg..

[86] van de Kelft E., Verleye G., van de Kelft A.S., Melis K., van Goethem J. (2017). Validation of Topographic Hybrid Single-Photon Emission Computerized Tomography with Computerized Tomography Scan in Patients with and without Nonspecific Chronic Low Back Pain. A Prospective Comparative Study. Spine J..

[87] Hsieh J.C., Belfrage M., Stone-Elander S., Hansson P., Ingvar M. (1995). Central Representation of Chronic Ongoing Neuropathic Pain Studied by Positron Emission Tomography. Pain.

[88] He B., Yang L., Wilke C., Yuan H. (2011). Electrophysiological Imaging of Brain Activity and Connectivity—Challenges and Opportunities. IEEE Trans. Biomed. Eng..

[89] Morton D., Jones A., Sandhu J. (2016). Brain Imaging of Pain: State of the Art. J. Pain Res..

[90] Peyron R., Laurent B., García-Larrea L. (2000). Functional Imaging of Brain Responses to Pain. A Review and Meta-Analysis (2000). Neurophysiol. Clin./Clin. Neurophysiol..

[91] Tracey I., Woolf C.J., Andrews N.A. (2019). Composite Pain Biomarker Signatures for Objective Assessment and Effective Treatment. Neuron.

[92] Apkarian A.V. (2004). Chronic Back Pain Is Associated with Decreased Prefrontal and Thalamic Gray Matter Density. J. Neurosci..

[93] Baliki M.N., Schnitzer T.J., Bauer W.R., Apkarian A.V. (2011). Brain Morphological Signatures for Chronic Pain. PLoS ONE.

[94] Seminowicz D.A., Wideman T.H., Naso L., Hatami-Khoroushahi Z., Fallatah S., Ware M.A., Jarzem P., Bushnell M.C., Shir Y., Ouellet J.A. (2011). Effective Treatment of Chronic Low Back Pain in Humans Reverses Abnormal Brain Anatomy and Function. J. Neurosci..

[95] Geha P.Y., Baliki M.N., Harden R.N., Bauer W.R., Parrish T.B., Apkarian A.V. (2008). The Brain in Chronic CRPS Pain: Abnormal Gray-White Matter Interactions in Emotional and Autonomic Regions. Neuron.

[96] Maeda Y., Kettner N., Sheehan J., Kim J., Cina S., Malatesta C., Gerber J., McManus C., Mezzacappa P., Morse L.R. (2013). Altered Brain Morphometry in Carpal Tunnel Syndrome Is Associated with Median Nerve Pathology. Neuroimage Clin..

[97] DaSilva A.F., Becerra L., Pendse G., Chizh B., Tully S., Borsook D. (2008). Colocalized Structural and Functional Changes in the Cortex of Patients with Trigeminal Neuropathic Pain. PLoS ONE.

[98] DeSouza D.D., Moayedi M., Chen D.Q., Davis K.D., Hodaie M. (2013). Sensorimotor and Pain Modulation Brain Abnormalities in Trigeminal Neuralgia: A Paroxysmal, Sensory-Triggered Neuropathic Pain. PLoS ONE.

[99] Gustin S.M., Wrigley P.J., Youssef A.M., McIndoe L., Wilcox S.L., Rae C.D., Edden R.A.E., Siddall P.J., Henderson L.A. (2014). Thalamic Activity and Biochemical Changes in Individuals with Neuropathic Pain after Spinal Cord Injury. Pain.

[100] Liu Y., Li J., Butzkueven H., Duan Y., Zhang M., Shu N., Li Y., Zhang Y., Li K. (2013). Microstructural Abnormalities in the Trigeminal Nerves of Patients with Trigeminal Neuralgia Revealed by Multiple Diffusion Metrics. Eur. J. Radiol..

[101] Iadarola M.J., Max M.B., Berman K.F., Byas-Smith M.G., Coghill R.C., Gracely R.H., Bennett G.J. (1995). Unilateral Decrease in Thalamic Activity Observed with Positron Emission Tomography in Patients with Chronic Neuropathic Pain. Pain.

[102] Hsieh J.-C., Ståhle-Bäckdahl M., Hägermark Ö., Stone-Elander S., Rosenquist G., Ingvar M. (1996). Traumatic Nociceptive Pain Activates the Hypothalamus and the Periaqueductal Gray: A Positron Emission Tomography Study. Pain.

[103] Petrovic P., Ingvar M., Stone-Elander S., Petersson M.K., Hansson P. (1999). A PET Activation Study of Dynamic Mechanical Allodynia in Patients with Mononeuropathy. Pain.

[104] Cauda F., Sacco K., D’Agata F., Duca S., Cocito D., Geminiani G., Migliorati F., Isoardo G. (2009). Low-Frequency BOLD Fluctuations Demonstrate Altered Thalamocortical Connectivity in Diabetic Neuropathic Pain. BMC Neurosci..

[105] Loggia M.L., Kim J., Gollub R.L., Vangel M.G., Kirsch I., Kong J., Wasan A.D., Napadow V. (2013). Default Mode Network Connectivity Encodes Clinical Pain: An Arterial Spin Labeling Study. Pain.

[106] Wasan A.D., Loggia M.L., Chen L.Q., Napadow V., Kong J., Gollub R.L. (2011). Neural Correlates of Chronic Low Back Pain Measured by Arterial Spin Labeling. Anesthesiology.

[107] Kishima H., Saitoh Y., Oshino S., Hosomi K., Ali M., Maruo T., Hirata M., Goto T., Yanagisawa T., Sumitani M. (2010). Modulation of Neuronal Activity after Spinal Cord Stimulation for Neuropathic Pain; H215O PET Study. Neuroimage.

[108] DosSantos M.F., Martikainen I.K., Nascimento T.D., Love T.M., Deboer M.D., Maslowski E.C., Monteiro A.A., Vincent M.B., Zubieta J.-K., DaSilva A.F. (2012). Reduced Basal Ganglia μ-Opioid Receptor Availability in Trigeminal Neuropathic Pain: A Pilot Study. Mol. Pain.

[109] Jones A.K.P., Kitchen N.D., Watabe H., Cunningham V.J., Jones T., Luthra S.K., Thomas D.G.T. (1999). Measurement of Changes in Opioid Receptor Binding in Vivo During Trigeminal Neuralgic Pain Using [^11^C]Diprenorphine and Positron Emission Tomography. J. Cereb. Blood Flow Metab..

[110] Klega A., Eberle T., Buchholz H.-G., Maus S., Maihöfner C., Schreckenberger M., Birklein F. (2010). Central Opioidergic Neurotransmission in Complex Regional Pain Syndrome. Neurology.

[111] Fukui S., Matsuno M., Inubushi T., Nosaka S. (2006). N-Acetylaspartate Concentrations in the Thalami of Neuropathic Pain Patients and Healthy Comparison Subjects Measured with 1H-MRS. Magn. Reson. Imaging.

[112] Grachev I.D., Fredrickson B.E., Apkarian V.A. (2000). Abnormal Brain Chemistry in Chronic Back Pain: An in Vivo Proton Magnetic Resonance Spectroscopy Study. Pain.

[113] Grachev I.D., Thomas P.S., Ramachandran T.S. (2002). Decreased Levels of N-Acetylaspartate in Dorsolateral Prefrontal Cortex in a Case of Intractable Severe Sympathetically Mediated Chronic Pain (Complex Regional Pain Syndrome, Type I). Brain Cogn..

[114] Pattany P.M., Yezierski R.P., Widerström-Noga E.G., Bowen B.C., Martinez-Arizala A., Garcia B.R., Quencer R.M. (2002). Proton Magnetic Resonance Spectroscopy of the Thalamus in Patients with Chronic Neuropathic Pain after Spinal Cord Injury. AJNR Am. J. Neuroradiol..

[115] Sorensen L., Siddall P.J., Trenell M.I., Yue D.K. (2008). Differences in Metabolites in Pain-Processing Brain Regions in Patients With Diabetes and Painful Neuropathy. Diabetes Care.

[116] di Pietro F., Macey P.M., Rae C.D., Alshelh Z., Macefield V.G., Vickers E.R., Henderson L.A. (2018). The Relationship between Thalamic GABA Content and Resting Cortical Rhythm in Neuropathic Pain. Hum. Brain Mapp..

[117] Janetzki L., Gussew A., Malessa R., Habenicht U., Reichenbach J.R., Strauß B., Borys C. (2016). Hirnmetabolische Veränderungen Bei Chronischem Rückenschmerz. Der Schmerz.

[118] Kameda T., Fukui S., Tominaga R., Sekiguchi M., Iwashita N., Ito K., Tanaka-Mizuno S., Konno S. (2018). Brain Metabolite Changes in the Anterior Cingulate Cortex of Chronic Low Back Pain Patients and Correlations Between Metabolites and Psychological State. Clin. J. Pain.

[119] Widerström-Noga E., Pattany P.M., Cruz-Almeida Y., Felix E.R., Perez S., Cardenas D.D., Martinez-Arizala A. (2013). Metabolite Concentrations in the Anterior Cingulate Cortex Predict High Neuropathic Pain Impact after Spinal Cord Injury. Pain.

[120] Guastella V., Mick G., Laurent B. (2008). Traitements Non Médicamenteux de La Douleur Neuropathique. Presse Med..

[121] Papuć E., Rejdak K. (2013). The Role of Neurostimulation in the Treatment of Neuropathic Pain. Ann. Agric. Environ. Med..

[122] Wolter T. (2014). Spinal Cord Stimulation for Neuropathic Pain: Current Perspectives. J. Pain Res..

[123] Hosobuchi Y., Adams J.E., Rutkin B. (1973). Chronic Thalamic Stimulation for the Control of Facial Anesthesia Dolorosa. Arch. Neurol..

[124] Owen S.L.F., Green A.L., Nandi D., Bittar R.G., Wang S., Aziz T.Z. (2006). Deep Brain Stimulation for Neuropathic Pain. Neuromodulation Technol. Neural Interface.

[125] Pereira E.A.C., Aziz T.Z. (2014). Neuropathic Pain and Deep Brain Stimulation. Neurotherapeutics.

[126] Bittar R.G., Kar-Purkayastha I., Owen S.L., Bear R.E., Green A., Wang S., Aziz T.Z. (2005). Deep Brain Stimulation for Pain Relief: A Meta-Analysis. J. Clin. Neurosci..

[127] Hamani C., Schwalb J.M., Rezai A.R., Dostrovsky J.O., Davis K.D., Lozano A.M. (2006). Deep Brain Stimulation for Chronic Neuropathic Pain: Long-Term Outcome and the Incidence of Insertional Effect. Pain.

[128] North R.B., Kidd D.H., Farrokhi F., Piantadosi S.A. (2005). Spinal Cord Stimulation versus Repeated Lumbosacral Spine Surgery for Chronic Pain: A Randomized, Controlled Trial. Neurosurgery.

[129] Cruccu G., Aziz T.Z., Garcia-Larrea L., Hansson P., Jensen T.S., Lefaucheur J.-P., Simpson B.A., Taylor R.S. (2007). EFNS Guidelines on Neurostimulation Therapy for Neuropathic Pain. Eur. J. Neurol..

[130] Kumar K., North R., Taylor R., Sculpher M., van den Abeele C., Gehring M., Jacques L., Eldabe S., Meglio M., Molet J. (2005). Spinal Cord Stimulation vs. Conventional Medical Management: A Prospective, Randomized, Controlled, Multicenter Study of Patients with Failed Back Surgery Syndrome (PROCESS Study). Neuromodulation Technol. Neural Interface.

[131] Kumar K., Taylor R.S., Jacques L., Eldabe S., Meglio M., Molet J., Thomson S., O’Callaghan J., Eisenberg E., Milbouw G. (2007). Spinal Cord Stimulation versus Conventional Medical Management for Neuropathic Pain: A Multicentre Randomised Controlled Trial in Patients with Failed Back Surgery Syndrome. Pain.

[132] Deer T.R., Levy R.M., Kramer J., Poree L., Amirdelfan K., Grigsby E., Staats P., Burton A.W., Burgher A.H., Obray J. (2017). Dorsal Root Ganglion Stimulation Yielded Higher Treatment Success Rate for Complex Regional Pain Syndrome and Causalgia at 3 and 12 Months: A Randomized Comparative Trial. Pain.

[133] Liem L., Russo M., Huygen F.J.P.M., van Buyten J.-P., Smet I., Verrills P., Cousins M., Brooker C., Levy R., Deer T. (2013). A Multicenter, Prospective Trial to Assess the Safety and Performance of the Spinal Modulation Dorsal Root Ganglion Neurostimulator System in the Treatment of Chronic Pain. Neuromodulation Technol. Neural Interface.

[134] Deer T.R., Grigsby E., Weiner R.L., Wilcosky B., Kramer J.M. (2013). A Prospective Study of Dorsal Root Ganglion Stimulation for the Relief of Chronic Pain. Neuromodulation Technol. Neural Interface.

[135] Peyron R., Garcia-Larrea L., Deiber M.P., Cinotti L., Convers P., Sindou M., Mauguière F., Laurent B. (1995). Electrical Stimulation of Precentral Cortical Area in the Treatment of Central Pain: Electrophysiological and PET Study. Pain.

[136] Maarrawi J., Peyron R., Mertens P., Costes N., Magnin M., Sindou M., Laurent B., Garcia-Larrea L. (2007). Motor Cortex Stimulation for Pain Control Induces Changes in the Endogenous Opioid System. Neurology.

[137] Fukazawa Y., Maeda T., Hamabe W., Kumamoto K., Gao Y., Yamamoto C., Ozaki M., Kishioka S. (2005). Activation of Spinal Anti-Analgesic System Following Electroacupuncture Stimulation in Rats. J. Pharmacol. Sci..

[138] Zhang G.G., Yu C., Lee W., Lao L., Ren K., Berman B.M. (2005). Involvement of Peripheral Opioid Mechanisms in Electroacupuncture Analgesia. EXPLORE.

[139] Johnson M. (2007). Transcutaneous Electrical Nerve Stimulation: Mechanisms, Clinical Application and Evidence. Rev. Pain.

[140] Garcia-Larrea L., Maarrawi J., Peyron R., Costes N., Mertens P., Magnin M., Laurent B. (2006). On the Relation between Sensory Deafferentation, Pain and Thalamic Activity in Wallenberg’s Syndrome: A PET-Scan Study before and after Motor Cortex Stimulation. Eur. J. Pain.

[141] Knotkova H., Hamani C., Sivanesan E., le Beuffe M.F.E., Moon J.Y., Cohen S.P., Huntoon M.A. (2021). Neuromodulation for Chronic Pain. Lancet.

[142] Sims-Williams H., Matthews J.C., Talbot P.S., Love-Jones S., Brooks J.C., Patel N.K., Pickering A.E. (2017). Deep Brain Stimulation of the Periaqueductal Gray Releases Endogenous Opioids in Humans. Neuroimage.

[143] Davis K.D., Taub E., Duffner F., Lozano A.M., Tasker R.R., Houle S., Dostrovsky J.O. (2000). Activation of the Anterior Cingulate Cortex by Thalamic Stimulation in Patients with Chronic Pain: A Positron Emission Tomography Study. J. Neurosurg..

[144] Jones S.E., Lempka S.F., Gopalakrishnan R., Baker K.B., Beall E.B., Bhattacharyya P., Huang X., Lin J., Chen J., Lowe M.J. (2021). Functional Magnetic Resonance Imaging Correlates of Ventral Striatal Deep Brain Stimulation for Poststroke Pain. Neuromodulation Technol. Neural Interface.

[145] See A.A.Q., King N.K.K. (2017). Improving Surgical Outcome Using Diffusion Tensor Imaging Techniques in Deep Brain Stimulation. Front. Surg..

[146] Pereira E.A.C., Green A.L., Bradley K.M., Soper N., Moir L., Stein J.F., Aziz T.Z. (2007). Regional Cerebral Perfusion Differences between Periventricular Grey, Thalamic and Dual Target Deep Brain Stimulation for Chronic Neuropathic Pain. Stereotact. Funct. Neurosurg..

[147] Mohseni H.R., Smith P.P., Parsons C.E., Young K.S., Hyam J.A., Stein A., Stein J.F., Green A.L., Aziz T.Z., Kringelbach M.L. (2012). MEG Can Map Short and Long-Term Changes in Brain Activity Following Deep Brain Stimulation for Chronic Pain. PLoS ONE.

[148] Bentley L.D., Duarte R.V., Furlong P.L., Ashford R.L., Raphael J.H. (2016). Brain Activity Modifications Following Spinal Cord Stimulation for Chronic Neuropathic Pain: A Systematic Review. Eur. J. Pain.

[149] Yearwood T., de Ridder D., Yoo H.B., Falowski S., Venkatesan L., Ting To W., Vanneste S. (2020). Comparison of Neural Activity in Chronic Pain Patients During Tonic and Burst Spinal Cord Stimulation Using Fluorodeoxyglucose Positron Emission Tomography. Neuromodulation Technol. Neural Interface.

[150] Pawela C.P., Kramer J.M., Hogan Q.H. (2017). Dorsal Root Ganglion Stimulation Attenuates the BOLD Signal Response to Noxious Sensory Input in Specific Brain Regions: Insights into a Possible Mechanism for Analgesia. Neuroimage.

[151] Peyron R., Faillenot I., Mertens P., Laurent B., Garcia-Larrea L. (2007). Motor Cortex Stimulation in Neuropathic Pain. Correlations between Analgesic Effect and Hemodynamic Changes in the Brain. A PET Study. Neuroimage.

[152] Kim J., Ryu S.B., Lee S.E., Shin J., Jung H.H., Kim S.J., Kim K.H., Chang J.W. (2016). Motor Cortex Stimulation and Neuropathic Pain: How Does Motor Cortex Stimulation Affect Pain-Signaling Pathways?. J. Neurosurg..

[153] Kara M., Özçakar L., Gökçay D., Özçelik E., Yörübulut M., Güneri S., Kaymak B., Akıncı A., Çetin A. (2010). Quantification of the Effects of Transcutaneous Electrical Nerve Stimulation With Functional Magnetic Resonance Imaging: A Double-Blind Randomized Placebo-Controlled Study. Arch. Phys. Med. Rehabil..

[154] Goto T., Saitoh Y., Hashimoto N., Hirata M., Kishima H., Oshino S., Tani N., Hosomi K., Kakigi R., Yoshimine T. (2008). Diffusion Tensor Fiber Tracking in Patients with Central Post-Stroke Pain; Correlation with Efficacy of Repetitive Transcranial Magnetic Stimulation. Pain.

[155] Diaz M.M., Caylor J., Strigo I., Lerman I., Henry B., Lopez E., Wallace M.S., Ellis R.J., Simmons A.N., Keltner J.R. (2022). Toward Composite Pain Biomarkers of Neuropathic Pain-Focus on Peripheral Neuropathic Pain. Front. Pain Res..

[156] Hoehne A., Behera D., Parsons W.H., James M.L., Shen B., Borgohain P., Bodapati D., Prabhakar A., Gambhir S.S., Yeomans D.C. (2013). A 18F-Labeled Saxitoxin Derivative for in Vivo PET-MR Imaging of Voltage-Gated Sodium Channel Expression Following Nerve Injury. J. Am. Chem. Soc..

[157] Lampert A., Bennett D.L., McDermott L.A., Neureiter A., Eberhardt E., Winner B., Zenke M. (2020). Human Sensory Neurons Derived from Pluripotent Stem Cells for Disease Modelling and Personalized Medicine. Neurobiol. Pain.

[158] Akin E.J., Alsaloum M., Higerd G.P., Liu S., Zhao P., Dib-Hajj F.B., Waxman S.G., Dib-Hajj S.D. (2021). Paclitaxel Increases Axonal Localization and Vesicular Trafficking of Nav1.7. Brain.

[159] Martinez A.L., Brea J., Barro M., Monroy X., Merlos M., Burgueño J., Loza M.I. (2021). Development of a Novel in Vitro Assay to Screen for Neuroprotective Drugs against Iatrogenic Neurite Shortening. PLoS ONE.

[160] Yang J.-L., Xu B., Li S.S., Zhang W.S., Xu H., Deng X.M., Zhang Y.Q. (2012). Gabapentin Reduces CX3CL1 Signaling and Blocks Spinal Microglial Activation in Monoarthritic Rats. Mol. Brain.

[161] Tung K.W., Behera D., Biswal S. (2015). Neuropathic Pain Mechanisms and Imaging. Semin. Musculoskelet. Radiol..

[162] Behera D., Shen B., James M.L. (2012). Radiolabeled Sigma-1 Receptor Ligand Detects Peripheral Neuroinflammation in a Neuropathic Pain Model Using PET-MRI. Proceedings of the 2012 World Molecular Imaging Congress.

[163] Cabañero D., Ramírez-López A., Drews E., Schmöle A., Otte D.M., Wawrzczak-Bargiela A., Huerga Encabo H., Kummer S., Ferrer-Montiel A., Przewlocki R. (2020). Protective Role of Neuronal and Lymphoid Cannabinoid CB2 Receptors in Neuropathic Pain. Elife.

[164] Saroz Y., Kho D.T., Glass M., Graham E.S., Grimsey N.L. (2019). Cannabinoid Receptor 2 (CB2) Signals via G-Alpha-s and Induces IL-6 and IL-10 Cytokine Secretion in Human Primary Leukocytes. ACS Pharmacol. Transl. Sci..

[165] Filipiuc L.E., Ababei D.C., Alexa-Stratulat T., Pricope C.V., Bild V., Stefanescu R., Stanciu G.D., Tamba B.-I. (2021). Major Phytocannabinoids and Their Related Compounds: Should We Only Search for Drugs That Act on Cannabinoid Receptors?. Pharmaceutics.

[166] Komorowska-Müller J.A., Schmöle A.-C. (2020). CB2 Receptor in Microglia: The Guardian of Self-Control. Int. J. Mol. Sci..

[167] Tamba B.I., Stanciu G.D., Urîtu C.M., Rezus E., Stefanescu R., Mihai C.T., Luca A., Rusu-Zota G., Leon-Constantin M.-M., Cojocaru E. (2020). Challenges and Opportunities in Preclinical Research of Synthetic Cannabinoids for Pain Therapy. Medicina (B Aires).

[168] Guindon J., Hohmann A.G. (2008). Cannabinoid CB2 Receptors: A Therapeutic Target for the Treatment of Inflammatory and Neuropathic Pain. Br. J. Pharmacol..

[169] Niu J., Huang D., Zhou R., Yue M.X., Xu T., Yang J., He L., Tian H., Liu X.H., Zeng J. (2017). Activation of Dorsal Horn Cannabinoid CB2 Receptor Suppresses the Expression of P2Y 12 and P2Y 13 Receptors in Neuropathic Pain Rats. J. Neuroinflamm..

[170] Malek N., Popiolek-Barczyk K., Mika J., Przewlocka B., Starowicz K. (2015). Anandamide, Acting via CB2 Receptors, Alleviates LPS-Induced Neuroinflammation in Rat Primary Microglial Cultures. Neural Plast..

[171] Guerrero-Alba R., Barragán-Iglesias P., González-Hernández A., Valdez-Moráles E.E., Granados-Soto V., Condés-Lara M., Rodríguez M.G., Marichal-Cancino B.A. (2019). Some Prospective Alternatives for Treating Pain: The Endocannabinoid System and Its Putative Receptors GPR18 and GPR55. Front. Pharmacol..

[172] Lauckner J.E., Jensen J.B., Chen H.Y., Lu H.C., Hille B., Mackie K. (2008). GPR55 Is a Cannabinoid Receptor That Increases Intracellular Calcium and Inhibits M Current. Proc. Natl. Acad. Sci. USA.

[173] Behera D., Jacobs K.E., Behera S., Rosenberg J., Biswal S. (2011). (18)F-FDG PET/MRI Can Be Used to Identify Injured Peripheral Nerves in a Model of Neuropathic Pain. J. Nucl. Med..

[174] Franc B.L., Acton P.D., Mari C., Hasegawa B.H. (2008). Small-Animal SPECT and SPECT/CT: Important Tools for Preclinical Investigation. J. Nucl. Med..

[175] Ahn B.-C. (2011). Applications of Molecular Imaging in Drug Discovery and Development Process. Curr. Pharm. Biotechnol..

[176] Matthews P.M., Coatney R., Alsaid H., Jucker B., Ashworth S., Parker C., Changani K. (2013). Technologies: Preclinical Imaging for Drug Development. Drug Discov. Today Technol..

[177] Bak M.S., Park H., Kim S.K. (2021). Neural Plasticity in the Brain during Neuropathic Pain. Biomedicines.

[178] Chung G., Kim C.Y., Yun Y.-C., Yoon S.H., Kim M.-H., Kim Y.K., Kim S.J. (2017). Upregulation of Prefrontal Metabotropic Glutamate Receptor 5 Mediates Neuropathic Pain and Negative Mood Symptoms after Spinal Nerve Injury in Rats. Sci. Rep..

[179] da Silva J.T., Seminowicz D.A. (2019). Neuroimaging of Pain in Animal Models: A Review of Recent Literature. Pain Rep..

[180] Hooker B.A., Tobon G., Baker S.J., Zhu C., Hesterman J., Schmidt K., Rajagovindan R., Chandran P., Joshi S.K., Bannon A.W. (2014). Gabapentin-Induced Pharmacodynamic Effects in the Spinal Nerve Ligation Model of Neuropathic Pain. Eur. J. Pain.

[181] Shen B., Behera D., James M.L., Reyes S.T., Andrews L., Cipriano P.W., Klukinov M., Lutz A.B., Mavlyutov T., Rosenberg J. (2017). Visualizing Nerve Injury in a Neuropathic Pain Model with [ ^18^ F]FTC-146 PET/MRI. Theranostics.

[182] Betti C., Starnowska J., Mika J., Dyniewicz J., Frankiewicz L., Novoa A., Bochynska M., Keresztes A., Kosson P., Makuch W. (2015). Dual Alleviation of Acute and Neuropathic Pain by Fused Opioid Agonist-Neurokinin 1 Antagonist Peptidomimetics. ACS Med. Chem. Lett..

[183] Witkowska E., Godlewska M., Osiejuk J., Gątarz S., Wileńska B., Kosińska K., Starnowska-Sokół J., Piotrowska A., Lipiński P.F.J., Matalińska J. (2022). Bifunctional Opioid/Melanocortin Peptidomimetics for Use in Neuropathic Pain: Variation in the Type and Length of the Linker Connecting the Two Pharmacophores. Int. J. Mol. Sci..

[184] Raffa R.B., Pergolizzi J.V., Taylor R., Ossipov M.H. (2018). Indirect-Acting Strategy of Opioid Action Instead of Direct Receptor Activation: Dual-Acting Enkephalinase Inhibitors (DENKIs). J. Clin. Pharm. Ther..

[185] Borsook D., Hargreaves R., Bountra C., Porreca F. (2014). Lost but Making Progress—Where Will New Analgesic Drugs Come From?. Sci. Transl. Med..

[186] Kadriu B., Ballard E.D., Henter I.D., Murata S., Gerlus N., Zarate C.A. (2020). Neurobiological Biomarkers of Response to Ketamine. Adv. Pharmacol..

[187] Kevadiya B.D., Ottemann B.M., Thomas M.B., Mukadam I., Nigam S., McMillan J., Gorantla S., Bronich T.K., Edagwa B., Gendelman H.E. (2019). Neurotheranostics as Personalized Medicines. Adv. Drug Deliv. Rev..

[188] Khanal M., Gohil S.V., Kuyinu E., Kan H.-M., Knight B.E., Baumbauer K.M., Lo K.W.-H., Walker J., Laurencin C.T., Nair L.S. (2018). Injectable Nanocomposite Analgesic Delivery System for Musculoskeletal Pain Management. Acta Biomater..

[189] Luan X., Sansanaphongpricha K., Myers I., Chen H., Yuan H., Sun D. (2017). Engineering Exosomes as Refined Biological Nanoplatforms for Drug Delivery. Acta Pharmacol. Sin..

[190] Choi H., Choi Y., Yim H.Y., Mirzaaghasi A., Yoo J.-K., Choi C. (2021). Biodistribution of Exosomes and Engineering Strategies for Targeted Delivery of Therapeutic Exosomes. Tissue Eng. Regen. Med..

[191] Banerjee A., Alves V., Rondão T., Sereno J., Neves Â., Lino M., Ribeiro A., Abrunhosa A.J., Ferreira L.S. (2019). A Positron-Emission Tomography (PET)/Magnetic Resonance Imaging (MRI) Platform to Track in Vivo Small Extracellular Vesicles. Nanoscale.

[192] Perets N., Betzer O., Shapira R., Brenstein S., Angel A., Sadan T., Ashery U., Popovtzer R., Offen D. (2019). Golden Exosomes Selectively Target Brain Pathologies in Neurodegenerative and Neurodevelopmental Disorders. Nano Lett..

[193] Lorenc T., Chrzanowski J., Olejarz W. (2020). Current Perspectives on Clinical Use of Exosomes as a Personalized Contrast Media and Theranostics. Cancers.

[194] Baron R., Maier C., Attal N., Binder A., Bouhassira D., Cruccu G., Finnerup N.B., Haanpää M., Hansson P., Hüllemann P. (2017). Peripheral Neuropathic Pain: A Mechanism-Related Organizing Principle Based on Sensory Profiles. Pain.

[195] Balzani E., Fanelli A., Malafoglia V., Tenti M., Ilari S., Corraro A., Muscoli C., Raffaeli W. (2021). A Review of the Clinical and Therapeutic Implications of Neuropathic Pain. Biomedicines.

